# Printing biohybrid materials for bioelectronic cardio-3D-cellular constructs

**DOI:** 10.1016/j.isci.2022.104552

**Published:** 2022-06-07

**Authors:** Paola Sanjuan-Alberte, Charlie Whitehead, Joshua N. Jones, João C. Silva, Nathan Carter, Simon Kellaway, Richard J.M. Hague, Joaquim M.S. Cabral, Frederico C. Ferreira, Lisa J. White, Frankie J. Rawson

**Affiliations:** 1 Regenerative Medicine and Cellular Therapies, School of Pharmacy, Biodiscovery Institute, University of Nottingham, University Park, Nottingham NG7 2RD, UK; 2 Department of Bioengineering and Institute for Bioengineering and Biosciences, Instituto Superior Técnico, Universidade de Lisboa, Av. Rovisco Pais, 1049-001 Lisbon, Portugal; 3 Associate Laboratory i4HB—Institute for Health and Bioeconomy, Instituto Superior Técnico, Universidade de Lisboa, Av. Rovisco Pais, 1049-001 Lisbon, Portugal; 4 Centre for Rapid and Sustainable Product Development, Polytechnic of Leiria, 2430-038 Marinha Grande, Portugal; 5 Department of Mechanical Engineering, University of Minnesota, Minneapolis, MN 55455, USA; 6 UCL Centre for Nerve Engineering, University College London, London WC1E 6BT, UK; 7 Centre for Additive Manufacturing, Faculty of Engineering, University of Nottingham, University Park, Nottingham NG7 2RD, UK

**Keywords:** Cardiovascular medicine, Bioelectronics, Materials science

## Abstract

Conductive hydrogels are emerging as promising materials for bioelectronic applications as they minimize the mismatch between biological and electronic systems. We propose a strategy to bioprint biohybrid conductive bioinks based on decellularized extracellular matrix (dECM) and multiwalled carbon nanotubes. These inks contained conductive features and morphology of the dECM fibers. Electrical stimulation (ES) was applied to bioprinted structures containing human pluripotent stem cell-derived cardiomyocytes (hPSC-CMs). It was observed that in the absence of external ES, the conductive properties of the materials can improve the contractile behavior of the hPSC-CMs, and this effect is enhanced under the application of external ES. Genetic markers indicated a trend toward a more mature state of the cells with upregulated calcium handling proteins and downregulation of calcium channels involved in the generation of pacemaking currents. These results demonstrate the potential of our strategy to manufacture conductive hydrogels in complex geometries for actuating purposes.

## Highlights

• Conductive biohybrid hydrogels were 3D bioprinted using the FRESH method
• MWCNTs increased the conductivity and fiber diameter of dECM hydrogels
• Bioactuating applications were explored on the bioprinted structures
• Material’s conductivity and external electrical stimulation improved cell contractility

## Graphical abstract

**Figure:**
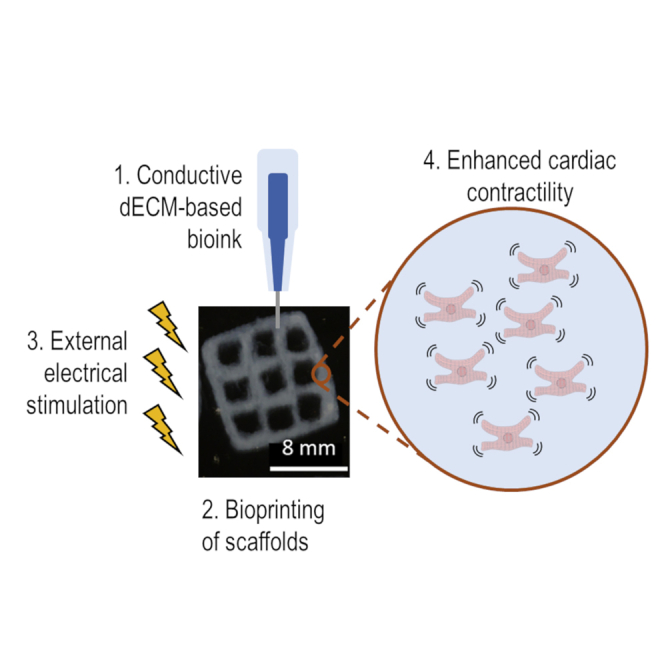

## Introduction

Cell-material interactions have traditionally been one of the main focuses of biomaterials and tissue engineering research. In the last decade, there has been an increased demand for smart and stimuli-responsive materials to provide additional control over material’s properties and cell fate (Distler et al., 2021). Enhanced functionalities are particularly important to improve the biomimicry of electroconductive tissues. For instance, it has now been widely accepted that conductive environments promote neural proliferation and differentiation (Garrudo et al., 2021; Wang et al., 2017). In addition, the development of bioelectronic systems and devices relies on the interface between biological and electroconductive systems.

Despite the increased popularity of synthetic conductive substrates for their ability to influence cell behavior, conductive natural biomaterials represent a better alternative because of their tissue-like characteristics and mechanical properties (Herrmann et al., 2021; Sanjuan-Alberte et al., 2021). There is also a mismatch in the conductive mechanism between electrically conductive synthetic substrates and ionically conductive tissues that needs further addressing and investigation (Casella et al., 2021). This mismatch can be minimized using conductive hydrogels, as these provide an ion-rich and wet physiological environment in a three-dimensional (3D) nanostructured and conductive network (Athukorala et al., 2021). The electrical conductivity of hydrogels can be increased by the incorporation of conductive micro- and nanofillers within the hydrogel matrix (Rastin et al., 2020). It has been hypothesized that the incorporation of the conductive materials bridges the insulating pore walls of the hydrogels, propagating the electrical signals and stimulating cell constructs evenly and uniformly (Spencer et al., 2019). The most commonly conductive nanofillers used include metallic nanoparticles (Baei et al., 2016), conductive polymers (Mawad et al., 2016) and carbon-based nanomaterials (Liu et al., 2017). In bioelectronics, conductive hydrogels have been explored for the development of wearable electronics (Chen et al., 2021), implantable devices (Hassarati et al., 2014) and sensing/actuating applications (Li et al., 2021).

There is a wide variety of natural biomaterials used for the development of conductive hydrogels. Decellularized extracellular matrix (dECM) materials have shown promise because functional and structural components of native ECM can be retained (Kim et al., 2020; Saldin et al., 2017), maintaining the biochemical cues that naturally interact with cells in a specific microenvironment (Agmon and Christman, 2016). Furthermore, dECM can be used in the composition of bioinks that subsequently allow the additive manufacturing of 3D structures (Shin et al., 2021). dECM-based hollow tubes and bifurcating structures resembling anatomical features such as blood vessels and airways have been 3D printed using the freeform embedding of suspended hydrogels (FRESH) extrusion method (De Santis et al., 2021). The variety of tissues from which dECM can be extracted determines the versatility and functionality of the bioprinted structures, where intrinsic cellular morphologies and functions can be reconstituted (Pati et al., 2014). There have been two recent reports on making dECM conductive with addition of carbon-based nanomaterials and subsequently merging them with cardiomyocytes (Bai et al., 2021; Tsui et al., 2021). However, neither included detailed analysis of the effect of the material in the electrical genotype of the cells. Such analysis is important as we have previously suggested that this is one of the most important functions to modulate when aiming at *in vitro* generation of mature cardiomyocytes (Vaithilingam et al., 2019).

In this work, a conductive bioink for the 3D bioprinting of structures has been developed, combining the electroconductive features of multiwalled carbon nanotubes (MWCNTs) with the biochemical and structural cues of dECM. Such inks have never been explored in 3D bioprinting. Initially, a general strategy to formulate inks and bioinks for FRESH extrusion bioprinting based on dECM extracted from several tissues was established. Once this was achieved, electroconductive hydrogels were formulated and characterized. Finally, and to explore the bioelectronic applications of this material, electrical stimulation to 3D printed structures containing cardiac cells was performed, evaluating the potential of the materials to regulate cardiac cells’ fate.

## Results and discussion

### Tissue decellularization and bioink formulation

As previously discussed, natural materials with enhanced conductivity have the potential to reduce the mismatch between electronic and biological components in bioelectronics and as such, dECM was selected as the main component of the conductive hydrogels developed in this work. Tissue decellularization was successfully achieved from three different organs: porcine small intestine submucosa (sisECM), porcine liver (lECM), and bovine cancellous bone (bECM). Our choice was based on the fact that these organs are readily available and the dECM extraction protocols have been validated previously (Hwang et al., 2017; Sawkins et al., 2013; Voytik-Harbin et al., 1998), providing versatile and robust protocols for bioink formulation and 3D bioprinting with these materials.

**Figure 1:**
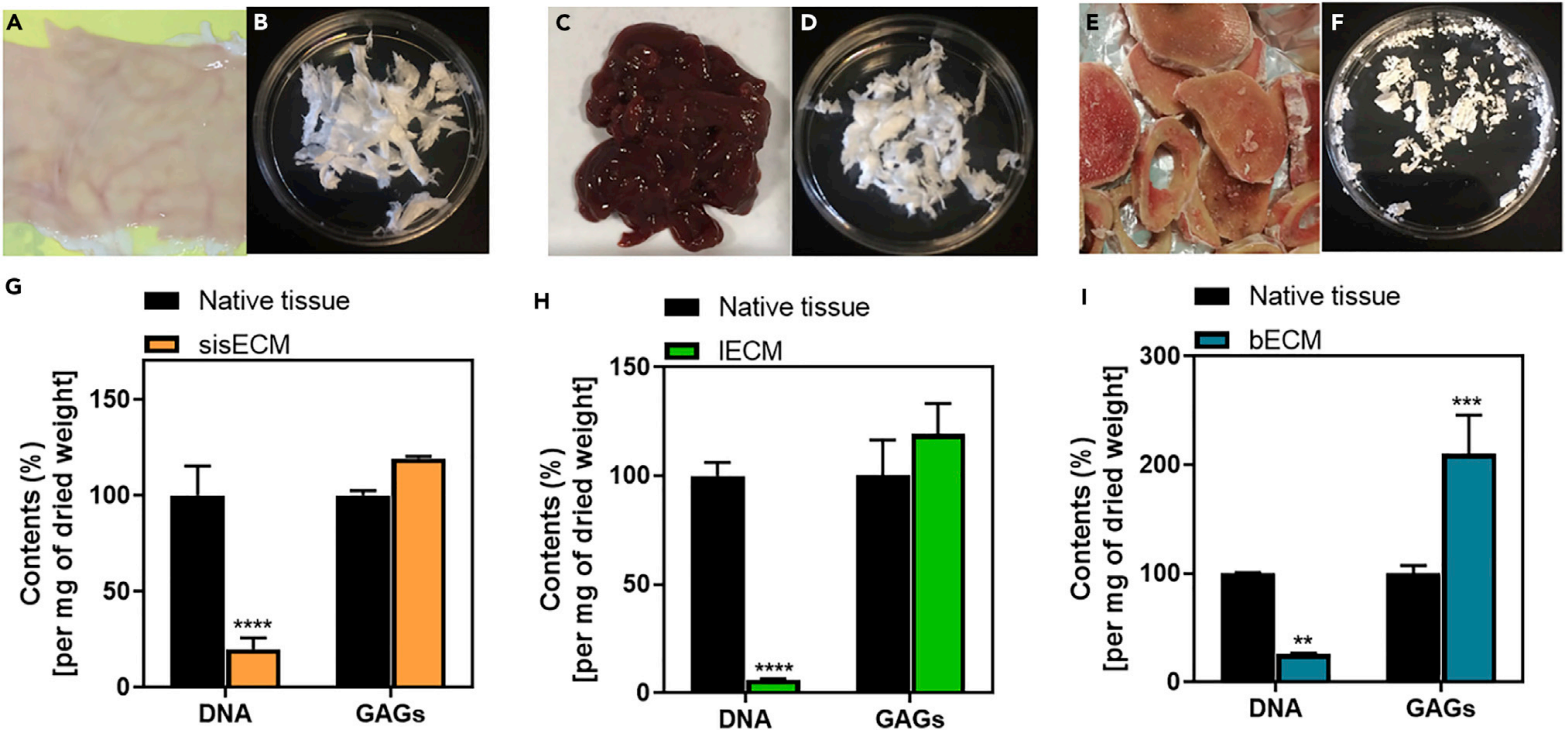
Decellularization and characterization process Three organs were decellularized for extracellular matrix (ECM) extraction. Porcine small intestine submucosa (sis) (A) before and (B) after decellularization (sisECM). Porcine liver (C) before and (D) after decellularization (lECM). Bovine bone (E) before and (F) after decellularization (bECM). Percentage of DNA and glycosaminoglycans (GAGs) present in native and decellularized ECM in (G) sis, (H) liver and (I) bone. Quantification was performed per mg of dried tissue. Composition of native tissue was assumed as 100% (n = 3).

Although we have not used native cardiac tissue ECM, we show later that bECM can solve some of the challenges we have raised, and it can be employed as a cell actuating biomaterial beyond cardiac tissue engineering applications. These materials have also been previously reported in the literature for applications in cardiac tissue repair and engineering (Ravi et al., 2012; Toeg et al., 2013). In the case of sisECM, cells were removed by mechanical delamination. The native tissue can be seen in Figure 1A and the results of the decellularization in Figure 1B. In the case of lECM, the extraction process involves enzymatic and chemical removal of the cells with detergents and images of native and lECM can be seen in Figures 1C and 1D, respectively. For bECM, the process included demineralisation and delipidation prior to enzymatic decellularization, with images of native and bECM shown in Figures 1E and 1F.

The main purpose of the decellularization process is to remove the native cells from these tissues while preserving the ECM structure and composition, which is not a trivial task (White et al., 2017). This is because residual cellular material can induce cytotoxic effects when ECM biomaterials are implanted *in vivo*. The amount of residual cellular material present in the decellularized tissues can be quantified by calculating the amount of DNA present in the dECM as remnant DNA can be directly correlated with residual cells within the dECM (Abaci and Guvendiren, 2020). Although the main goal is to remove cells effectively, the ECM structure and components such as collagen and glycosaminoglycans (GAGs) need to be preserved during the decellularization process. The quantification of structural molecules is therefore crucial to evaluate the quality of the decellularized products.

In the case of DNA quantification, the DNA content of the dECM is significantly reduced after the decellularization process as expected, corresponding to 19.84% for sisECM, 6.05% for lECM and 25.98% for bECM (Figures 1G–1I, respectively). The lower DNA percentage was obtained in lECM as a combination of enzymatic and chemical methods to remove cells are usually more effective. From these results we concluded that the tissues have been effectively decellularized and the structural molecules have been preserved.

In all tissues, the GAG content was relatively high when compared to native tissues, indicating that the decellularization process did not cause any structural damages to the extracted dECM (Figures 1G–1I). The GAG quantification was normalized per mg of dried tissue and the contents of the native tissues were assumed as 100%. Because the native tissues still preserve the cellular material, the weight of the ECM is more diluted, causing the GAG content of the dECM to be >100%, similar to that described previously (Pati et al., 2014).

### FRESH extrusion bioprinting

**Figure 2:**
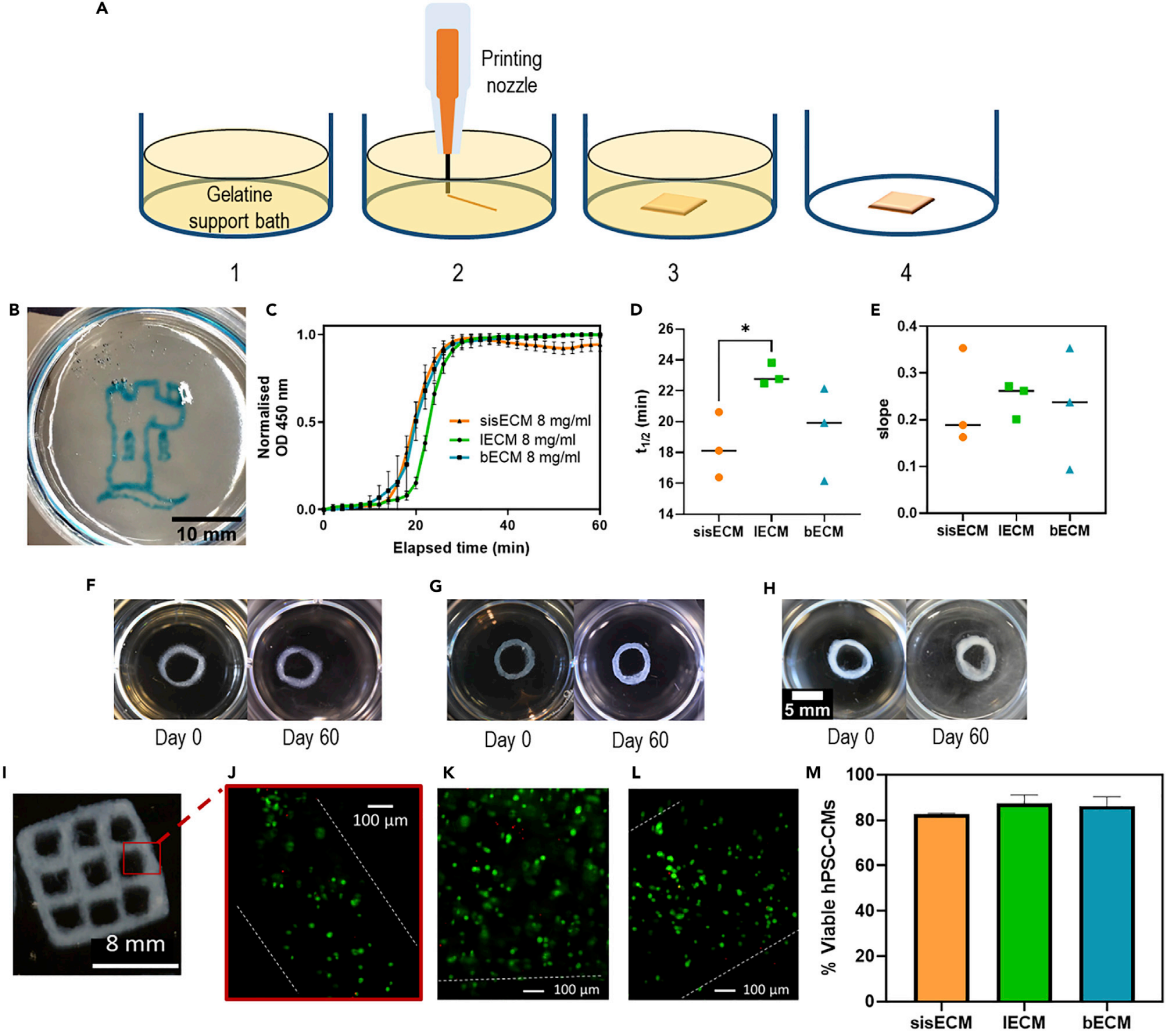
FRESH bioprinting of dECM hydrogels (A) Schematic representation of the process of FRESH extrusion printing of ECM hydrogels. 1. A thermo-reversible support bath formed by gelatine microparticles is used as a substrate. 2. Extrusion printing of cold decellularized ECM (dECM) takes place inside the gelatine bath. 3. *In situ* gelation of printed dECM structures at room temperature. 4. Structure is released when the temperature is increased to 37°C. (B) Printed bECM structure following the FRESH extrusion method. Scale bar 10 mm. (C) Normalized turbidimetric gelation kinetics of sisECM, lECM and bECM at 450 nm (n = 3). Determination of the kinetics parameters (D) t_1/2_ and (E) slope. Five-layered printed 6 mm diameter rings of (F) sisECM, (G) lECM, and (H) bECM and their appearance on day 0 and day 60 after printing. (I) Example of a 10 × 10 mm bECM 3D bioprinted scaffolds and inset of (J) fluorescence image of bioprinted hPSC-CMs in bECM after live/dead staining. Representative fluorescence microscopy images of bioprinted hPSC-CMs in (K) sisECM and (L) lECM after live/dead staining. (M) Percentage of viable cells after bioprinting using the different bioinks (n = 3, error bars represent +/-1 standard deviation fo the mean). The dotted white line indicates the edge of the structures. Images were taken on day 7 after bioprinting. See also Figures S1–S5.

The FRESH extrusion printing method was used in the manufacturing of complex dECM structures. This method consists of the printing of materials inside a gelatine slurry and is commonly used for the bioprinting of hydrogels as it allows the deposition and cross-linking of soft biomaterials while avoiding their collapse and deformation during the printing process (Hinton et al., 2015) (Figure 2A). An example of the complexity of the structures manufactured using the bECM ink can be seen in Figure 2B. The advantage of the FRESH technique over the reported conventional extrusion bioprinting methods of dECM-based bioinks (Jang et al., 2016) is that no photo-crosslinking with UV is required.

The intrinsic properties of the gels vary between tissuetypes, therefore, the gelation kinetics of the different dECMs was evaluated to determine the time required for the structures to fully gelate after printing. A turbidimetric evaluation was used on the different tissues, as changes in the turbidity of the solutions provide a rapid and reproducible way of monitoring the collagen fibrillogenesis (Lam et al., 2020). As it can be seen in Figure 2C, the three distinct phases of fibrillogenesis can be observed: a lag phase, an exponential growth phase, and a plateau phase. For the different dECM types, the turbidity profile was similar in all cases. The previous graphs were fitted into sigmoidal curves (Figure S1) to determine the kinetics parameters of t_1/2_, corresponding to the time needed to reach 50% of the maximum absorbance values, and the slope of the curves, corresponding to the rate of fibrillogenesis. Values of t_1/2_ of sisECM, lECM and bECM were 18.1, 22.8, and 19.9 min, respectively, with a statistically significant difference between sisECM and lECM. For the slope, the data dispersion was bigger and no significant differences were observed. From this data, the faster gelation kinetics corresponded to sisECM, followed by bECM and lECM and we concluded that we could safely remove the gelatine support bath 30–40 min after the printing of the structures.

To assess the stability of the printed structures, 6 mm rings and squares were incubated in PBS for 60 days. As it can be seen in Figures 2F–2H, for all the three tissues, the structures remained stable during the 60 days. Additional images can be found in Figure S2.

To explore the bioelectronic applications of our materials, human induced pluripotent stem cell-derived cardiomyocytes (hPSC-CMs) were selected because of their electrogenic nature. These cells were differentiated from hPSCs following previous protocols (Mosqueira et al., 2018) resulting in purities >95% of hPSC-CMs for the different batches (Figure S3). To optimize the bioprinting parameters and assess the shear stress effects on cell viability, suspended hPSC-CMs in culture media were extruded using different needles (200, 400, and 600 μm) and printing pressures (1, 2, 5, and 10 psi) (Figure S4). There were not observed major differences between the different conditions, with cell viabilities between 75–90% in all cases in contrast to the 90% viability observed at the controls (Figure S5). Some reduction in cell viability can be expected since hPSC-CMs are subjected to additional stress during the bioprinting process. Values > 75% of viability are considered acceptable in bioprinting and are similar to other hPSC-CMs bioprinting studies (Maiullari et al., 2018; Sánchez et al., 2020).

Once it was established that the bioprinting process does not affect in great measure the hPSC-CMs viability, hPSC-CMs were incorporated into the dECM bioinks. 10 mm^2^ meshes were bioprinted using the different dECM materials (Figure 2I). Calcein-AM and ethidium homodimer staining was performed 7 days after bioprinting of the structures and the results showed that high viability was maintained on the different bioinks (Figures 2J-2L). It is important to note that although some cells started to elongate, overall the spheroidal structure of the hPSC-CMs was maintained after bioprinting. This could be because of the lack of mechanical support offered by the dECM or cell-cell interactions, as hPSC-CMs are encapsulated in a 3D structure. Achieving elongated cells is currently one of the main challenges in bioprinting of cardiac tissues (Soltan et al., 2019). The percentage of viable hPSC-CMs was similar for the three types of dECM with the highest viability observed in lECM (87.3%) followed by bECM (86.2%) and sisECM (82.6%) (Figure 2M).

### Electroconductive dECM-based hydrogels

Multifunctional features were introduced in the dECM, forming bioelectronic hydrogels when interfaced with cells by incorporating MWCNTs based on our previous work where we have seen that composites containing MWCNTs can affect the phenotype of hPSC-CMs (Vaithilingam et al., 2019). MWCNTs also present several advantages over other metal-based nanofillers and conductive polymers. When processed in the correct conditions, the visualization of cells within the structure is possible, biocompatible and can be easily biofunctionalized (Goding et al., 2018).

To discard any cytotoxic effects associated to the incorporation of the MWCNTs, sisECM, lECM, and bECM hydrogels containing 1 mg mL^−1^ MWCNTs (sisECM-MWNCTs, lECM-MWCNTs, and bECM-MWCNTs, respectively) and a suspension of hPSC-CMs were casted using 5 mm molds. Live/dead staining confirmed that most of the cells in the structures remained viable and that the incorporation of MWCNTs to the hydrogels did not induce noticeable cytotoxic effects in the hPSC-CMs (Figure S6).

**Figure 3:**
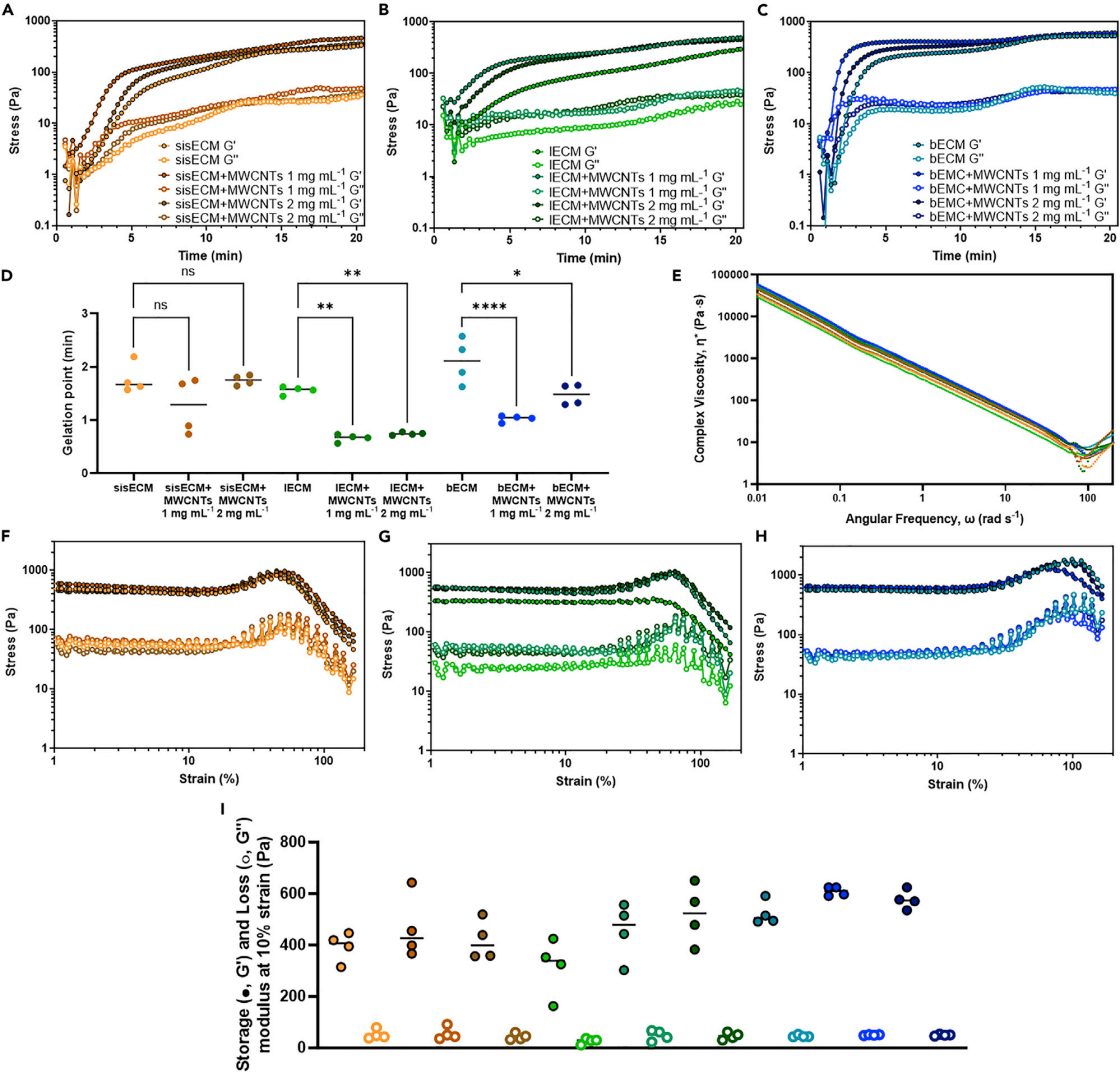
Rheological behavior of the different dECM inks Gelation kinetics showing the storage (G′) and loss moduli (G″) over time of (A) sisECM, (B) lECM, and (C) bECM with and without MWCNTs at 1 mg mL^−1^ and 2 mg mL^−1^. (D Gelation point and (E) complex viscosity of the different materials. Strain-sweeps of (F) sisECM, (G) lECM, and (H) bECM pre-gels with MWCNTs at 1 mg mL^−1^ and 2 mg mL^−1^ concentrations. (I) Values of storage (G′) and loss (G″) modulus at 10% strain. Four samples were analyzed for each hydrogel composition (n = 4, error bars represent +/-1 standard deviation fo the mean) from the same batch. See also Figure S6.

Rheological characterisation of the different inks was performed to assess whether the effect of MWCNTs in the gelation and viscoelastic properties of the dECM materials could affect the printability of the inks. Initially, gelation kinetics were evaluated by increasing the temperature to 37°C during a time-sweep rheometric test to trigger the collagen fibrillogenesis process. As expected, both the storage and loss moduli of all samples increased, with rapid onset of gelation upon ramping the temperature to 37°C, indicating that the materials were transitioning to the gel state (Figures 3A–3C). From these, it can be seen that bECM describes a more obvious sigmoidal curve than sisECM and lECM. The gelation point of plain sisECM, lECM and bECM was similar, with values of 1.63, 1.59, and 1.89 min, respectively (Figure 3D). In the case of sisECM, the addition of the MWCNTs at concentrations of 1 mg mL^−1^ and 2 mg mL^−1^, did not significantly affect the gelation point of the inks. However, in lECM and bECM, the addition of the MWCNTs caused a decrease in the gelation time. Similar observations were also made in studies using carboxylic and hydroxyl-functionalized MWCNTs (MWCNTs-COOH and MWCNTs-OH) in glycol/chitosan gels (Ravanbakhsh et al., 2019) and MWCNTs-COOH in polysaccharide-based hydrogels (Wang et al., 2021). One hypothesis to explain this observation could be that the carboxylic groups of the MWCNTs are contributing to the generation of additional bonds in the hydrogel.

A frequency-sweep test was performed on all materials. Shear-thinning flow behavior enables inks to be extrudable and reduce the shear forces exerted in the printing nozzles, and thus, a frequency-sweep test was performed in the materials. In all cases, the complex viscosity decreased linearly (Figure 3E), indicating a shear-thinning behavior. At high values of frequency (>50%) some irregularities can be seen in the graph, indicating some damage to the materials. In addition, a strain-sweep test was also performed to evaluate the linear-viscoelastic (LVE) limit on the different materials once they transitioned to the gel state. In sisECM, a linear strain-stress behavior up to 21% strain was observed (Figure 3F), where the addition of the MWCNTs did not seem to have a significant effect. In lECM the linear stress-strain region reached 44.5% strain (Figure 3G), and in the presence of MWCNTs, this value decreased to 25%. In bECM, linearity was observed up to 28% strain, with no noticeable differences among samples containing MWCNTs (Figure 3H). All samples exhibited decreasing G′ and G″ values after approximately 50% strain, leading to catastrophic failures.

G′ and G″ values from the different materials were compared at 10% strain, corresponding to the LVE region. G′ values of sisECM corresponded to 419 Pa (Figure 3I), and the addition of MWCNTs to the hydrogels did not seem to induce any changes to the material behavior. In the case of lECM, G′ increases with the concentration of MWCNTs present in the gel, with values of 352 Pa, 443 Pa, and 478 Pa for lECM, lECM + MWCNTs 1 mg mL^−1^, and lECM + MWCNTs 2 mg mL^−1^, respectively (Figure 3I). A similar trend was also observed in bECM, where G′ values were 491 Pa, 591 Pa, and 576 Pa in bECM, bECM + MWCNTs 1 mg mL^−1^, and bECM + MWCNTs 2 mg mL^−1^ (Figure 3I). The magnitude of G″ was similar in all samples and no noticeable differences were seen.

Despite all the materials exhibiting a similar rheological behavior and presenting minor differences, it was not possible to process the sisECM and lECM inks containing MWCNTs in the bioprinter. We observed with these inks that continuous clogging of the needle tip was being produced, limiting considerably the printability of structures. For this reason, in subsequent bioprinting experiments, bECM inks were selected. The printability (Pr) of these inks was evaluated on printed meshes with 10 × 10 mm. The semiquantification of the Pr, was calculated based on previous works from Equation 2 (Abassi et al., 2012), where the acceptable range of Pr was established at 0.9–1.1. In our case, the Pr of the bECM, bECM + MWCNTs 1 mg mL^−1^, and bECM + MWCNTs 2 mg mL^−1^ was <1, but the values were within the acceptable printability region, and no significant differences were observed between them (Figure S7). The printed structures remained stable for up to 30 days (Figure S8).

**Figure 4:**
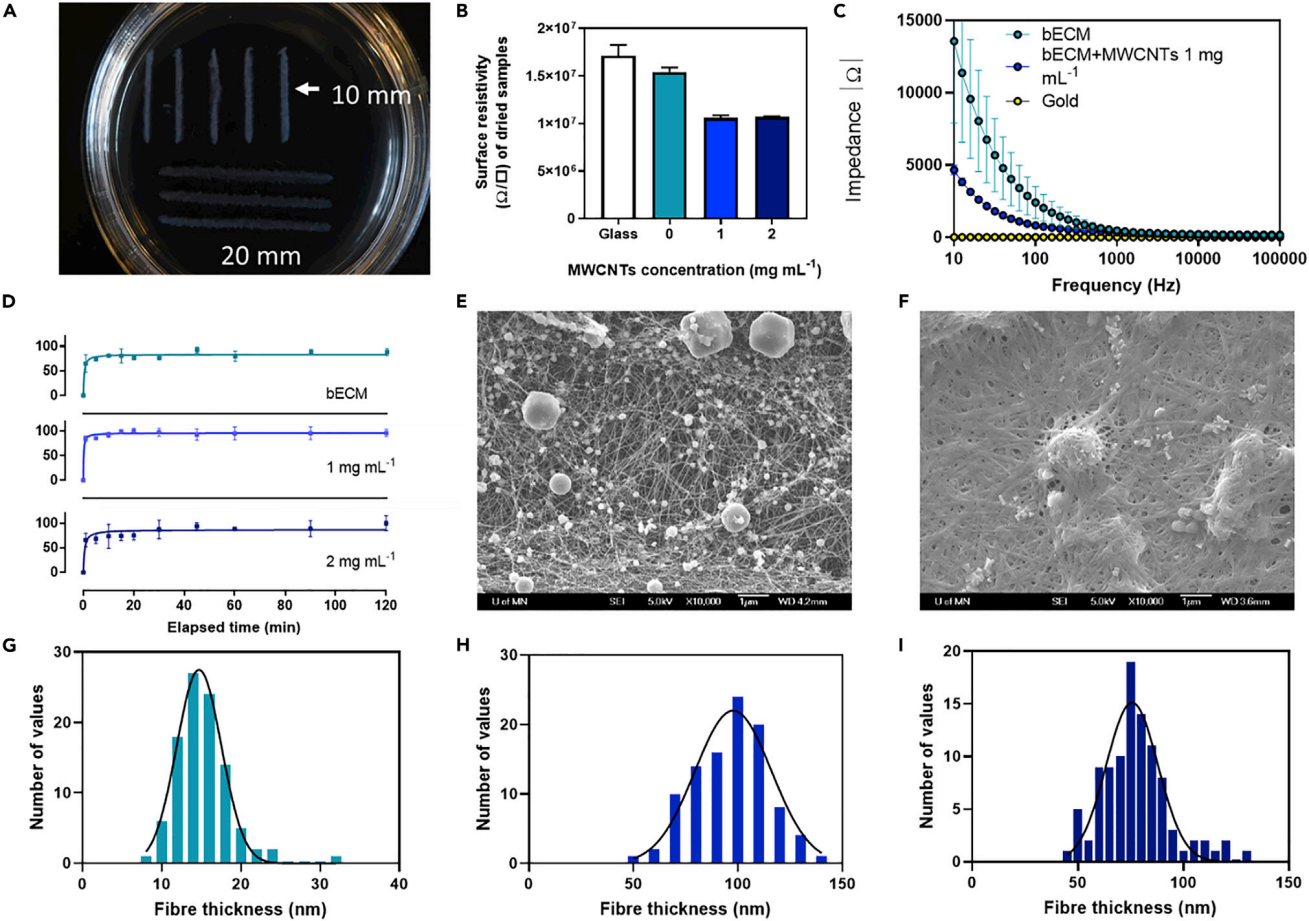
Characterization of conductive dECM hydrogels (A) 10 and 20 mm length printed structures used in the determination of surface resistivity. (B) Surface resistivity of dried printed samples (n = 3, error bars represent +/-1 standard deviation of the mean). (C) Electrochemical impedance spectroscopy of bECM and bECM + MWCNTs 1 mg mL^−1^ compared to bare gold (n = 3). (D) Swelling degree determination of printed bECM at increasing concentrations of MWCNTs (n = 3). Scanning electron microscopy (SEM) images of (E) bECM and (F) bECM + MWCNTs 1 mg mL^−1^. Histogram analysis and Gaussian distribution of fiber thickness taken from SEM images of (G) bECM, (H) bECM + MWCNTs 1 mg mL^−1^ and (I) bECM + MWCNTs 2 mg mL^−1^ (n = 100). See also Figures S7–S10.

We then determined the resistivity and impedance values of the hydrogels using a 4-probe method and electrochemical impedance spectroscopy (EIS), respectively. For the determination of the resistivity values on the dry materials, 10 and 20 mm lines were printed and dried (Figure 4A). The results of the surface resistivity calculation indicate that the addition of the MWCNTs to the structures contributed to decrease the surface resistivity values, from 154 MΩ/sq in bECM to 106.1 MΩ/sq in bECM + MWCNTs 1 mg mL^−1^ and to 106.9 MΩ/sq in bECM + MWCNTs 2 mg mL^−1^ (Figure 4B). All resistivity values were below the controls (glass surfaces) (171 MΩ/sq).

These results indicated MWCNTs decreases the resistivity of the materials. However, hydrogels are complex environments with diverse electronic properties. To investigate this, EIS measurements were conducted on hydrated samples, comparing bECM with bECM + MWCNTs 1 mg mL^−1^ and a fully conductive material (gold). On the samples containing MWCNTs, a decrease of an order of magnitude in values of impedance can be observed (Figure 4C), similarly to previous observations in gelatine methacrylate hydrogels containing MWCNTs (Izadifar et al., 2018; Sanjuan-Alberte et al., 2021). For instance, at 100 Hz, the values of impedance corresponded to 2408.7 in bECM, 823.1 in bECM + MWCNTs 1 mg mL^−1^ and 4.8 in gold, confirming that the addition of MWCNTs also contributes to increasing the conductivity of materials in wet conditions and the potential of this material in bioelectronics.

Further characterization was performed on the bECM, bECM + MWCNTs 1 mg mL^−1^, and bECM + MWCNTs 2 mg mL^−1^ printed constructs to investigate any other contributions of the MWCNTs to the hydrogel structure. The swelling degree indicated a rapid hydration of the bECM lyophilised hydrogels, reaching a plateau phase after 20–30 min incubation (Figure 4D). In samples containing MWCNTs the recovery of the hydrogel structure followed the same trend.

Printed samples were also dehydrated using a critical point drying method to preserve their ultrastructure and imaged by SEM. These images showed significant differences in the morphology and thickness of the bECM fibers with and without MWCNTs (Figures 4E and 4F). Histogram analysis of the fiber thickness of the different samples was performed, where the average thickness of bECM fibers corresponded to 15.16 nm, in contrast to the 96.17 and 77.46 nm measured in bECM + MWCNTs 1 mg mL^−1^ and bECM + MWCNTs 2 mg mL^−1^, respectively. Interestingly, MWCNTs were not observed at these concentrations in contrast to images at lower MWCNTs concentrations of 0.2 mg mL^−1^, where bECM fibers and MWCNTs are easily distinguishable (Figure S9). In addition, the typical collagen structure with a marked d-period was only observed at the higher MWCNTs concentrations. This suggests that an interaction between the bECM fibers and MWCNTs might be occurring at higher concentrations, leading to higher fiber diameters and reinforcing the strength of hydrogels as seen in Figure 3I. It can also be noted from the SEM images that some salts are also present in the hydrogels. Higher magnification images can be seen in Figure S10.

### Effects of electrical stimulation (ES) on hPSC-CMs

We then investigated the combination of biochemical cues, electrical conductivity, mechanical properties and ES on hPSC-CMs contractility, and maturity. Previous studies demonstrated that hydrogels containing MWCNTs led to improved neuronal differentiation and morphology, and the differences were magnified under ES (Imaninezhad et al., 2018).

**Figure 5:**
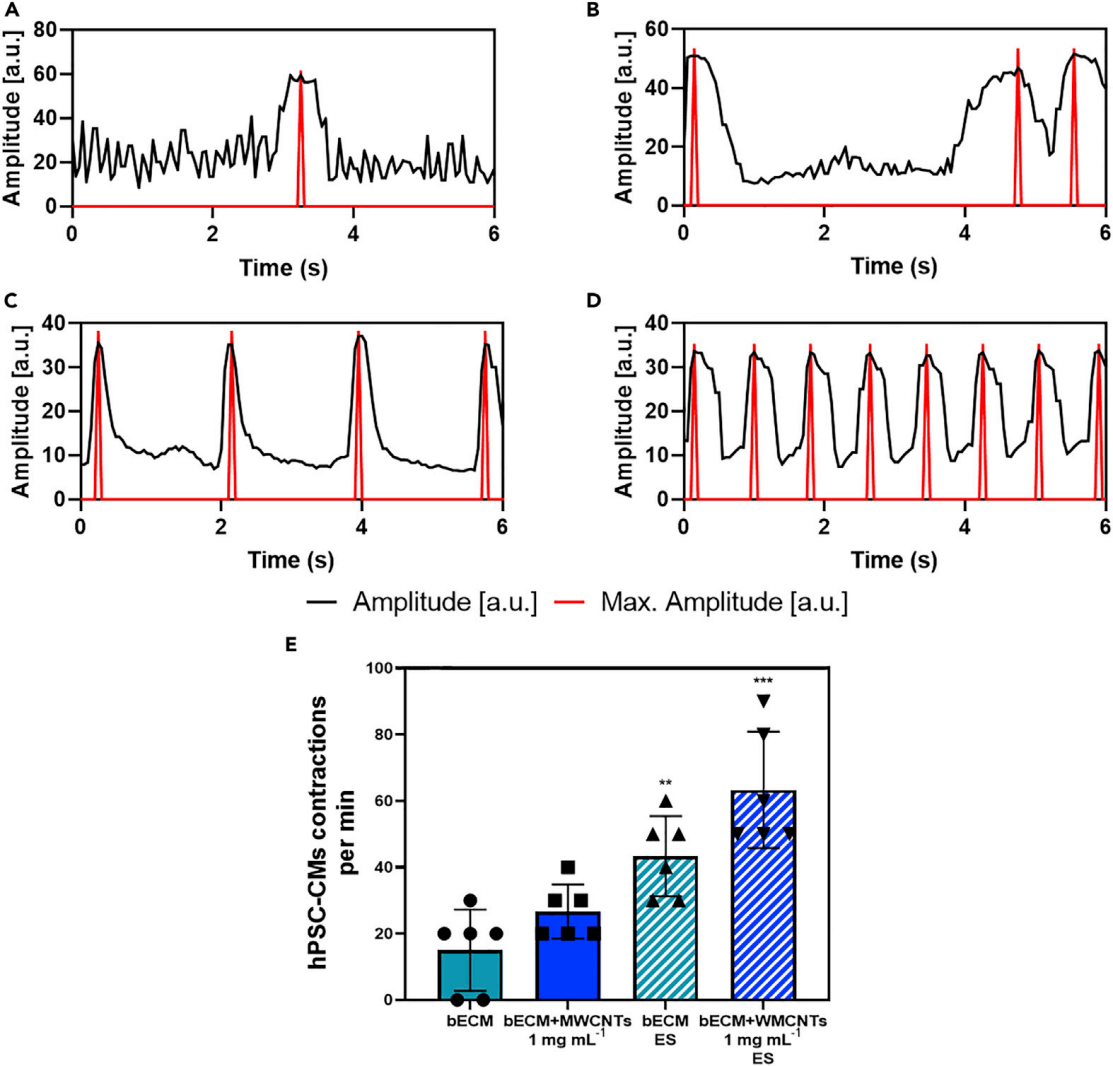
hPSC-CMs contractility assessment Time-dependent changes in autonomous contractile behavior of hPSC-CMs determined using the analytical tool Myocyter (v1.3), where contractions translate to positive going transients with an arbitrary unit (a.u.), of (A) bECM, (B) bECM + MWCNTs 1 mg mL^−1^, (C) bECM under electrical stimulation (ES), and (D) bECM + MWCNTs 1 mg mL^−1^ under ES. (E) Contraction rate of hPSC-CMs per minute on the different samples (n = 6, error bars represent +/-1 standard deviation of the mean) (∗∗p = 0.0024, ∗∗∗p = 0.0009). See also Figure S11, Videos S1, S2, S3, and S4.

Bioprinted structures containing hPSC-CMs were subjected to a regime of 2 h per day for 5 days of ES (a square wave, 2 V to −2 V, 1 Hz) and the contractile behavior of the cells was analyzed. In bECM and no ES, the spontaneous contractions of the cardiomyocytes were either not observed or very sporadic (Figures 5A, S11, and Video S1). The contractility of bioprinted cells improved slightly in bECM + MWCNTs 1 mg mL^−1^ hydrogels; however, contractions still exhibit an erratic pattern typical of arrhythmic cardiac tissues (Figure 5B and Video S2). Under the application of ES, the contractions of the hPSC-CMs were more defined and rhythmic (Figures 5C, 5D, Video S3) and the contraction rate was significantly higher than in structures that were not subjected to ES (Figure 5E and Video S4), which was particularly enhanced in the presence of MWCNTs reaching physiological values. These results demonstrate two findings: (1) the material’s conductive properties alone can support improvements in hPSC-CMs contractile behavior (despite no significant differences were seen between bECM and bECM + MWCNTs) and; (2) such improvement on the contractile behavior can be significantly enhanced when ES is applied. We tentatively hypothesize that this could be because of a combined effect of electrochemical and structural cues provided by the MWCNTs, which mean the structures act as bipolar electrodes localizing electric field effects (Qin et al., 2020).

Video S1. hPSC-CMs contraction rate on bECM and no electrical stimulation, related to Figure 5

Video S2. hPSC-CMs contraction rate on bECM + MWCNTs 1 mg mL-1 and no electrical stimulation, related to Figure 5

Video S3. hPSC-CMs contraction rate on bECM after electrical stimulation, related to Figure 5

Video S4. hPSC-CMs contraction rate on bECM + MWCNTs 1 mg mL-1 after electrical stimulation, related to Figure 5

We proceeded to evaluate any effects in the genotype of the cells using markers linked to cell maturity, morphology, electrophysiology, and calcium handling behavior by RT-PCR analysis. In this case, RT-PCR was selected for this analysis as it can provide more precise information on the developmental state of cells and because fluorescence markers are difficult to visualize in cells encapsulated in 3D hydrogels.

**Figure 6:**
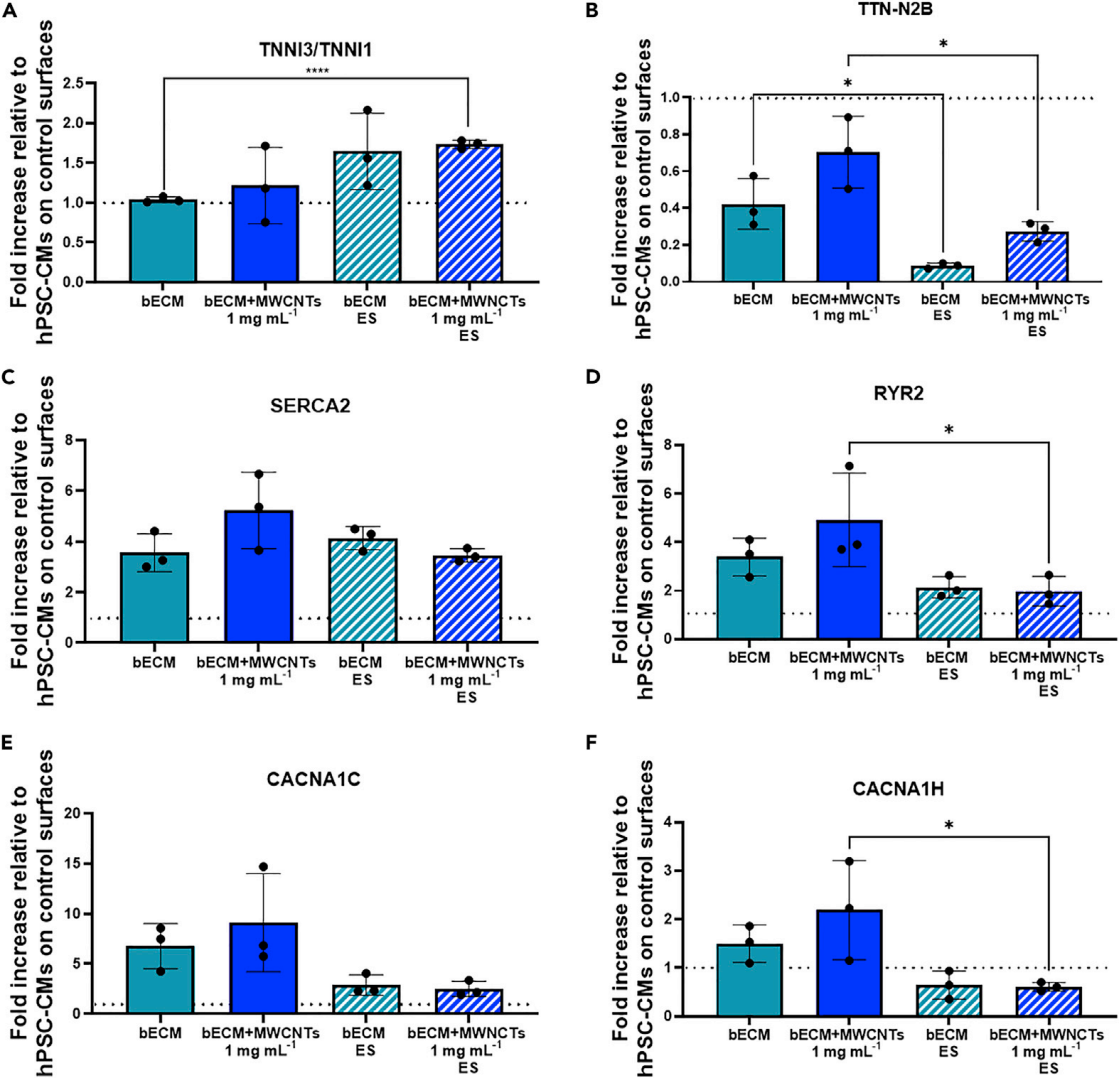
RT-qPCR analysis of the hPSC-CMs on bECM and bECM + MWCNTs 1 mg mL^−1^ with and without ES (A) TNNI3/TNNI1, (B) TTN-N2B, (C) SERCA2, (D) RYR2, (E) CACNA1C, and (F) CACNA1H gene expressions are normalized against the housekeeping gene GAPDH and presented as fold-change levels relative to hPSC-CMs on control surfaces. Error bars represent +/-1 standard deviation of the mean of three (n = 3) independent experiments.

An indicative marker for the maturation state of hPSC-CMs was obtained from the developmentally controlled and irreversible genetic switch in the TNNI gene. The TNNI isoform switch has been frequently used as a quantitative maturation signal for hPSC-CMs (Bedada et al., 2014). The TNNI1 gene (ssTnI) is expressed in the sarcomeres of fetal and neonatal hearts, which is then replaced by the TNNI3 (cTnI) isoform. The ratio between TNNI3/TNNI1 can provide an indication on the maturation state of the cells. The level of TNNI3/TNNI1 expression was similar in bioprinted cells in bECM than in controls (Figure 6A), indicating that the levels of the TNNI switch were not significantly different. This was also the case of bioprinted hPSC-CMs in bECM + MWCNTs without ES. For bioprinted cells in bECM under ES, there is a slight increase in the TNNI3/TNNI1 expression values; however, this was not enough to be significantly different. This was not the case of hPSC-CMs bioprinted in bECM + MWCNTs under ES, where a significant difference can be observed when compared to bECM. This result suggests that the combination of the conductivity and ES of the materials may increase the maturation level of the cells. Additional maturation studies based on alternative morphological and genetic markers need to be performed to confirm this hypothesis.

TTN-N2B was selected as a marker of cell morphology and function, as this is an elastic protein expressed in the sarcomeres of hPSC-CMs. The RT-PCR results showed that this gene was downregulated in all cases when compared to controls (Figure 6B). We hypothesize that this could be because of the nontypical morphology of the hPSC-CMs encapsulated in the hydrogels, where a spherical morphology was observed (Figures 2 and S7). Future strategies will be aimed at achieving higher control over the nano- and micro-topographies of the hydrogels to enhance the morphology of the cells.

The expression of the calcium-handling genes SERCA2 and RYR2, involved in the excitation-contraction coupling, was then evaluated. Previous studies reported an increased expression of these genes in hPSC-CMs when encapsulated in 3D hydrogels of cardiac dECM (Leonard et al., 2018). In our study, these genes were upregulated in all cases (Figures 6C and 6D); however, no major differences were observed in the bioprinted structures subjected to ES. Similar results were previously reported and it has been hypothesized that although ES could induce electrophysiological alterations similar to native development, they may not promote all aspects of the complex process of electrophysiological maturation (Baumgartner et al., 2015).

Expression of electrophysiological markers of hPSC-CMs was also evaluated through the CACNA1C and CACNA1H gene expression, coding for L-type and T-type calcium channels, respectively. L-type Ca^2+^ currents contribute to the shape of the cardiac action potential, and its regulation plays an important role in cardiac excitability and contractility (Uzun et al., 2016). The CACNA1C gene was overexpressed in all cases (Figure 6E), and its expression was more accentuated when no ES was applied, similarly to the case of SERCA2 and RYR2. T-type Ca^2+^ currents are more present in pace making heart cells but absent in the myocardium. In the absence of ES, the CACNA1H gene was upregulated; however, when external ES was applied, this stimulation was predominant for the pacing of the cells, and thus, this gene was downregulated when ES was applied (Figure 6F). Additional analysis of the electrophysiology of the bioprinted tissues, such as fractional shortening, could be performed to support these findings; however, these are difficult to implement in the present study because of the size limitations of our constructs.

### Conclusions

In this work, a strategy to develop novel and versatile bioinks for FRESH extrusion bioprinting is proposed with potential applications in bioelectronics. The main component of this bioink is a decellularized extracellular matrix, providing a suitable environment for cell growth with tailored biochemical signaling. 3D bioprinted structures were successfully manufactured, encapsulating hPSC-CMs while maintaining high cell viability. To further explore the sensing/actuating potential of this material, MWCNTs were dispersed in the hydrogel matrix, enhancing the electrical properties and structure of printed dECM hydrogels. Electrical stimulation was then applied and our results showed that the combination of an electrically conductive material with external electrical stimulation can drive contraction rates similar to physiological conditions. This demonstrates the potential of this material to be used in the development of smart scaffolds for biosensing/actuating applications. RT-PCR results indicated a significant improvement in the maturity markers of hPSC-CMs and downregulation of L-type Ca^2+^ currents, typically observed in pacemaker heart cells, which can be an indication that the profile of the cells is more aligned to those found in the myocardium.

### Limitations of the study

Despite the evaluation of the genetic information of the cells in this study, their morphological features were not optimal, exhibiting a spherical shape instead of the typical rod shape of the cardiomyocytes probably because of the lack of resolution of the extrusion bioprinting technique. Further development in the 3D bioprinting technology, combining techniques that can accurately reproduce the macro-, micro-, and nano-architectures of tissues are required to achieve higher biomimicry. In the future it would also be of interest to evaluate cardiac dECM; however, this is out of the scope of this work, because we wanted to evaluate the conservation of ECM proteins instead of tissue-specific or homologous ECM.

In addition, *in vivo* studies could provide further information on the behavior of these materials in physiological conditions and whether they maintain their bioelectrical and mechanical properties after implanting. Although dECM is a material commonly used for surgical reconstruction of the heart, future clinical evaluation of its combination with conductive materials could enable further bioactuating and biosensing capabilities.

## STAR★Methods

### Key resources table

**Table:** 

REAGENT or RESOURCE	SOURCE	IDENTIFIER
**Antibodies**		

Monoclonal Anti-TNNI3 antibody produced in mouse	Sigma Aldrich	WH0007137M4
Goat anti-Mouse secondary antibody IgG	Abcam	ab6785

**Biological samples**		

Decellularized porcine small intestine submucosa	This manuscript	N/A
Decellularized porcine liver	This manuscript	N/A
Decellularized bovine cancellous bone	This manuscript	N/A

**Chemicals, peptides, and recombinant proteins**		

Peroxyacetic acid, ca. 35wt.% sol. in diluted acetic acid, stabilized (PAA)	Acros Organics	257755000
Trypsin solution from porcine pancreas	Sigma Aldrich	T4424
Ethylenediaminetetraacetic acid (EDTA)	Thermo Scientific	J62948A1
Triton™ X-100	Sigma Aldrich	X100
Deoxycholic acid	Sigma Aldrich	D2510
Papain from *Carica papaya*	Sigma Aldrich	10108014001
Pepsin from porcine gastric mucosa	Sigma Aldrich	P7012
Multi-walled carbon nanotubes (MWCNTs) (>95%, OD: 10–20 nm)	US Nano	US4306
LifeSupport™ for FRESH	Allevi	FF-0002
RPMI 1640 Medium	Thermo Fischer	11875119
B-27™ Supplement (50X), custom	Thermo Fischer	0080085SA
Y-27632 ROCK inhibitor	Tocris	1254
Foetal bovine serum (FBS)	Sigma Aldrich	F4135
bisBenzimide Hoechst 33258	Sigma Aldrich	B2883
Acetoxymethyl (AM) calcein solution	Sigma Aldrich	C1359
Ethidium homodimer I	Sigma Aldrich	E1903

**Critical commercial assays**		

Quant-iT™ Picogreen® assay kit	Thermo Fischer	P7589
Hydroxyproline Assay Kit (DMMB)	Sigma Aldrich	MAK008
RNeasy Mini kit	Qiagen	74106
cDNA Reverse Transcription Kit	Thermo Fischer	4374966
iQ Universal SYBR Green Supermix	Bio-Rad	1708880
FRESH Kit	Allevi	N/A

**Experimental models: Cell lines**		

hPSC Line	Chris Denning’s Lab (University of Nottingham)	REBL-PAT

**Oligonucleotides**		

See Table S1 for details on the primers used	StabVida	N/A

**Software and algorithms**		

ImageJ open-source software	N/A	https://imagej.nih.gov/ij/
GraphPad Prism	GraphPad Software, San Diego, California USA	www.graphpad.com
MYOCYTER v1.3	Dr Tobias Jung, Nuthetal, Germany	http://www.scyrus.de/page9.html

**Other**		

Fisnar F5200N.2 3-Axis Inline Gantry Robot	Fisnar, Germantown, Wisconsin, USA	https://www.fisnar.com/products/robotics/benchtop-robots/f5200n-2-compact-gantry-benchtop-robot/
Ultimus V High Precision Dispenser	Nordson Corporation, Westlake, Ohio, USA	https://www.nordson.com/en/divisions/efd/products/fluid-dispensing-systems/ultimus-v-high-precision-dispenser
Physical MCR 301 Modular Compact Rheometer	Anton Paar, Graz, Austria	https://www.anton-paar.com/sg-en/products/details/rheometer-mcr-102-302-502/
Keithley 2400 Graphical Series SMU	Tektronix, Beaverton, Oregon, USA	https://www.tek.com/en/products/keithley/source-measure-units/2400-graphical-series-sourcemeter
FRA32 Electrochemical impedance spectroscopy module	Metrohm Autolab, Herisau, Switzerland	https://www.metrohm.com/en_nl/products/f/ra32/fra32m.html
Samdri-780a Critical Point Drier	Tousimis, Maryland, USA	N/A
JSM-7100F Thermal field emission electron microscope	Jeol, Tokyo, Japan	https://www.jeolbenelux.com/JEOL-BV-News/jsm-7100f-thermal-field-emission-electron-microscope
AFG1000 Arbitrary/Function Generator	Tektronix, Beaverton, Oregon, USA	https://www.tek.com/en/products/signal-generators/arbitrary-function-generator/afg1000

### Resource availability

#### Lead contact

Further information and requests for resources and reagents should be directed to and will be fulfilled by the lead contact, Frankie Rawson (Frankie.Rawson@nottingham.ac.uk).

#### Materials availability

The dECM types generated in this study are available at Lisa White’s lab, University of Nottingham. No further unique reagents where generated in this study.

### Method details

#### Tissue decellularization and characterisation

All the tissues used in this work were harvested from 12- to 24-month-old animals from an EU-certified butcher and/or abattoir.

For the preparation of the small intestine submucosa extracellular matrix (sisECM), porcine small intestines were abundantly washed with water and intestinal contents were discarded. Using forceps and a razor blade, the intestine was cut and opened and the serosa, mucosa and muscularis layers were removed, leaving behind the submucosa layers (Badylak et al., 1989). The resultant tissue was rinsed with water and cut into smaller pieces, which were then washed in distilled water (dH_2_O) under agitation for 2 h. Tissue depyrogenation was carried out in peracetic acid (PAA) 0.1% in a 24:1 dH_2_O/ethanol solution for 2 h under agitation and washed with phosphate buffer saline (PBS) and dH_2_O.

Decellularized liver extracellular matrix (lECM) was prepared from a previously frozen porcine liver. Frozen tissue was sliced into 3–4 cm cuts and washed in dH_2_O to remove any excess blood in a mechanical shaker. This solution was then replaced with a 0.02% Trypsin/0.05% EDTA solution in PBS, incubated at 37°C for 1 h. A 3% Triton-X-100 in dH_2_O was then used for 1 h at room temperature. Finally, tissues were washed with 4% deoxycholic acid (w/v) in dH_2_O (Loneker et al., 2016). In between solution exchanges, tissues were thoroughly washed with dH_2_O and pressed between pieces of mesh to aid in cell lysis (Loneker et al., 2016). PAA depyrogenation was carried out following the aforementioned procedure.

Decellularized bone extracellular matrix (bECM) was prepared following previous protocols (Alom et al., 2018; Sawkins et al., 2013) from cancellous bone segments. Briefly, bone segments were washed with 0.1% w/v gentamicin in PBS. Following this, segments were immersed in liquid nitrogen and ground in a bladed grinder. Bone granules were demineralised in 0.5 N HCl (25 mL per gram of bone) for 24 h at room temperature under stirring and washed with dH_2_O. Lipids were extracted using a 1:1 mixture of chloroform/methanol (30 mL per gram of bone) for 1 h at room temperature and washed with methanol and dH_2_O. Enzymatic decellularization was performed using a 0.05% trypsin/0.02% EDTA solution in PBS at 37°C for 24 h under constant agitation. The decellularized bone was then washed in an equal volume of PBS at 37°C for 24h under constant agitation, and then rinsed in dH_2_O.

In all cases, the decellularized materials were lyophilised, milled and stored at – 20°C for further use.

Quantification of DNA and sulphated glycosaminoglycans was performed as described previously (Chiti et al., 2021). Briefly, the determination of decellularization efficiency was based on quantification of dry weight double-stranded DNA (dsDNA). Concentrations of dsDNA were measured using a Quant-iT™ Picogreen® assay kit following the instructions provided by the supplier. Sulphated GAGs were determined using a 1,9 dimethyl-methylene blue (DMMB) hydroxyproline assay. A papain buffer consisting on 100 mM of Na_2_HPO_4_, 10 mM of EDTA, 10 mM of glycine and 125 μg mL^−1^ papain was used to digest dECM at a concentration of 10 mg mL^−1^. A blank papain buffer was used as a negative control and diluent for the assay. A total of 50 μL of samples were mixed with 200 μL of DMMB solution (0.03 M sodium formate, 0.046 M DMMB, ethanol [0.5% v/v] and formic acid [0.2% v/v] in a 96-well plate. The standard curve ranged from 0 to 100 μg mL^−1^ chondroitin-4-sulphate. Absorbance was measured at 525 and 595 nm. Samples are analyzed by taking the difference of the readings between absorbance at 525 and 595nm (OD_525_–OD_595_).

#### dECM ink formulation and printing

Lyophilised dECM material was enzymatically digested in a solution of 1 mg mL^−1^ porcine pepsin in 0.01 N HCl under stirring for 48 h at room temperature to obtain dECM digest with a final concentration of 10 mg mL^−1^. These solutions were stored at −20°C when not immediately used. To induce the gelation of the dECM digests (pH = 2–3), a neutralisation buffer consisting of 0.1 M NaOH and PBS was added to the dECM digest following previous protocols (Alom et al., 2018) and kept at 4°C to prevent the spontaneous gelation before the 3D printing process was carried out, with a final concentration of 8 mg mL^−1^. Prior to printing, this material was loaded into a 3cc QuantX™ syringe barrel with a 27G straight cannula blunt end dispensing tip (Fisnar). This pre-hydrogel solution was kept at 4°C during the preparation and printing process to prevent its gelation before the printing process was carried out.

In samples containing -COOH functionalised multi-walled carbon nanotubes (MWCNTs) these were dispersed at a concentration of 5–10 mg mL^−1^ in the neutralisation buffer and mixed with the dECM digests as described previously to reach a final concentration of 1–2 mg mL^−1^ in the dECM pre-hydrogel solution. A schematic of this can be seen in Figure S1.

For the 3D bio-/printing, a custom microextrusion-based 3D printing system was used as described previously (Joung et al., 2018). Briefly, the system consisted of a three-axis dispensing robot (Fisnar F5200N) and a pneumatic dispensing unit (Ultimus V) interfaced with a personal computer. Printing speeds for the dECM bioinks ranged 0.8–2 mm s^−1^ and the printing pressures used ranged 0.1–0.5 psi. Coordinates of the dispensing robot were programmed using G-code commands. The gelatine support bath was prepared using an FRESH Kit and LifeSupport™ for FRESH in a Well-plate.

After printing the different structures, printed samples were kept at room temperature for 20 min to induce the gelation of the dECM and then incubated at 37°C for 30 min to melt the gelatine support bath. Once the gelatine melted, it was carefully aspirated and structures were kept in PBS or culture media.

#### Gelation kinetics and stability over time of printed dECM structures

A BioTek ELx800 plate reader interfaced with a personal computer incorporated with the Gen5 2.06 data analysis software was used to measure the absorbance of the dECM hydrogels over time. The absorbance was measured at 450 nm (at room temperature) and measurements were repeatedly undertaken for 60 min at 2 min intervals on the neutralised digested dECM. Values were normalised to allow further comparison between the different tissues. Data were normalised using Equation 1 and the turbidity profile was fitted to a sigmoid curve to calculate the t_1/2_, the time needed to reach 50% of the maximum turbidity absorbance value, and slope. The slope determined the speed of gelation. Statistical significance was calculated using a paired t-test.

(Equation 1) $$Normalised\;Absorbance\;\left(NA\right)=\frac{A-A_{0}}{A_{max}-A_{0}}\;$$

Where is A is absorbance.

For stability studies, 6 mm side squares and 6 mm diameter rings with three layers each were printed using the aborementioned conditions and incubated in PBS. Images of the structures were obtained on days 0, 3, 5, 7, 10, 15, 20, 30 and 60 post-printing.

#### hPSC-CMs differentiation and bioink formulation

A REBL-PAT hPSC line was derived from a skin punch biopsy from a male subject. Procedures of isolation, culture, differentiation and dissociation are described elsewhere (Mosqueira et al., 2018). Dissociation of cells took place 6–8 weeks after the differentiation process. Cell culture media consisted in basal RPMI medium supplemented with B27, Y-27632 ROCK inhibitor (20 μM) and 10% fetal bovine serum (FBS). The medium was changed after 24 h to RPMI/B27 medium. Early hPSC-CMs were incorporated into the bioinks by adding the cell suspension (1/5 of the final volume of the bioink) at an approximate concentration of 0.five to one million cells mL^−1^ to the neutralised dECM pre-hydrogels. Bioprinting was carried out using the aforementioned parameters.

#### hPSC-CMs purity assessment

hPSC-CMs were fixed in 2% paraformaldehyde for 30 min at room temperature and permeabilised with 0.1% Triton X-100 for 8 min at room temperature. Non-specific binding was blocked with 4% FBS in PBS for 1 h at room temperature. Cells were then immunostained with a monoclonal primary antibody against TNNI3 pat a concentration of 1:1000 in PBS overnight at 4°C. A solution of 0.05% of Tween 20 was used to wash the samples and a solution of goat anti-Mouse secondary antibody IgG was added (1:1000) and incubated for 2 h at room temperature. Samples were washed with the previous Tween 20 solution and exposed to Hoechst 33258 (5 μg mL^−1^). Fluorescence images were taken on a fluorescence microscope (Leica DMI 3000B equipped with Nikon-AcT1 software). The purity of differentiated cells was analyzed by calculating the ratio of hPSC-CMs (cells stained both with Hoechst and TNNI3) vs the total population of cells by fluorescence microscopy. Two different batches of cells were analyzed in triplicate and two images were quantified per sample. Statistical significance was calculated using a t-test (n = 3, n = 2; ±SD).

#### hPSC-CMs viability assays

Viability studies were performed on bioprinted 10 × 10 mm squared meshes using the different bioinks. On day 7, structures were carefully washed in PBS and incubated for 30 min in 1 μM acetoxymethyl (AM) calcein solution in PBS to stain viable cells (green) and dead cells (red) were stained with 5 μM ethidium homodimer I in PBS. Fluorescence images were taken in the aforementioned fluorescence microscope. Images were processed using ImageJ. To assess the effects of shear stress on hPSC-CMs viability during the printing process, different pressures (1, 2, 5 and 10 psi) and needle diameters (200 μm, 400 μm and 600 μm OD) were studied on cell suspensions in culture media and compared to control conditions (hPSC-CMs cultured on a polystyrene well-plate). Experiments were performed in triplicate.

#### Rheological characterisation

A Physical MCR 301 instrument was used for the rheological characterisation of the pre-gels. For this time sweep analysis, 200 μL of the samples were added to a pre-cooled to 4°C Peltier plate. A Peltier hood was used to keep the humidity conditions stable during the measurements. The geometry used was a 25 mm diameter parallel plate at a working distance of 0.4 mm. After samples were in place, the temperature of the Peltier plate was increased to 37°C. Angular frequency and amplitude were kept constant at 1 rad s^−1^ and 1%, respectively. Readings were taken once every 15 s for 20 min to obtain storage (G′) and loss (G″) modulus. The gelation point was then calculated, corresponding to the moment where G’ = G″.

Amplitude sweep analyses were performed immediately after the previous test. The angular frequency remained constant at 1 rad s^−1^ and the oscillatory strain ramped from 1–200%. The temperature was kept constant at 37°C.

For each test, four samples were analyzed per material (n = 4). Statistical significance was calculated using one-way ANOVA.

#### Semi-quantification of printability

Semi-quantification of the printability of bECM, bECM + MWCNTs 1 mg mL^−1^ and bECM + MWCNTs 2 mg mL^−1^ was calculated following previously described protocols (Ouyang et al., 2016). Briefly, 10 × 10 mm square meshes were 3D printed with the different materials and the perimeter of the square gaps of the mesh was calculated using ImageJ. The printability was then calculated using the following formula:

(Equation 2) $$Printability\;\left(Pr\right)=\frac{\pi }{4}\cdot \frac{1}{C}=\frac{L^{2}}{16A}$$

Where *C* corresponds to circularity, *L* to the length of the perimeter and *A* to the area of the perimeter.

#### Electrochemical characterisation of conductive hydrogels

A Keithley 2450 source meter connected to a four-probe station was used for the conductivity measurements of dried samples. 10 mm lines of bECM, bECM + MWCNTs 1 mg mL^−1^ and bECM + MWCNTS 2 mg mL^−1^ were 3D printed and dried at room temperature. A fixed current of 10 μA was applied through the outer probes and the resulting potential was measured at the inner probes. Readings were taken once every 1 s for 30 min. From the stable region of the graphs, the sheet resistance was then calculated (n = 3, ±SD).

Electrochemical impedance spectroscopy (EIS) was performed on a PGSTAT potentiostat including the FRA32M module and interfaced with a personal computer including the NovaLab software. The electrochemical cell consisted of two sputter-coated gold electrodes separated by a 2 mm gap in a two-electrode configuration at room temperature. Hydrogels were casted using molds with 2 mm height and 10 mm diameter and sandwiched between the gold electrodes. Frequencies applied ranged between 10–100000 Hz and the amplitude was 0.01 V_RMS_.

#### Swelling degree

For the assessment of the swelling degree, 6 mm diameter rings of the different materials were 3D printed and immersed in liquid nitrogen followed by lyophilisation. The initial weight of the dried materials was recorded (W_d_) and then samples were immersed in PBS at 37°C. At specific time points (1, 5, 10, 15, 20, 30, 45, 60, 90 and 120 min), the water excess was carefully removed using Kimtech paper and the weight of the samples was recorded (W_w_) (n = 3). The swelling percentage was calculated using the formula below:

(Equation 3) $$Swelling\;\%=\frac{W_{w}-W_{d}}{A_{d}}\;\times 100$$

#### Scanning electron microscopy imaging

6 mm diameter rings were printed and mounted on filter paper to facilitate the manipulation of the samples. Before SEM imaging, samples were dried using a Samdri-780a critical point drier. 100% ethanol was used before CO_2_ addition. After drying, samples were mounted on an aluminum pin stub with carbon tape prior to coating with a 5 nm layer of iridium on a Leica EM ACE 600. SEM images were obtained on a field-emission scanning electron microscope (FEG-SEM JOEL 7100F) in secondary electron mode with an acceleration voltage of 5 kV and a working distance of 10 mm. Fiber diameters were calculated by measuring the diameter of 100 fibers per sample and histograms were produced using GraphPad Prism v8.4.3.

#### Electrical stimulation and measurement of cellular contractility

Bioprinted structures of bECM and bECM + MWCNTs 1 mg mL^−1^ containing hPSC-CMs at a concentration of 1 million cells mL^−1^ were electrically stimulated for five days during 2 h. Electrical stimulation was provided using an AFG1022 arbitrary function generator. Stimulation parameters consisted of a square wave with an amplitude of 4 V (2 V to −2 V) at a frequency of 1 Hz. Quantification of the contraction rate was calculated from videos of the hPSC-CMs on the different samples with and without electrical stimulation using the MYOCYTER v1.3 macro for ImageJ. This macro enables the scaling of the time-dependent changes of pixel intensity in subsequent frames of the recorded cardiomyocytes, enabling the depiction of cellular contractility as positive amplitudes on an arbitrary scale. Data extraction was performed according to the developer’s instructions (Grune et al., 2019) (n = 3, n = 2, ±SD). Statistical significance was calculated using a t-test.

#### Gene expression analysis

hPSC-CMs were dissociated from bioprinted structures and total RNA was isolated using the RNeasy Mini kit following the manufacturer’s guidelines. RNA concentrations were calculated using a NanoVue Plus spectrophotometer (GE Healthcare). RNA was reverse transcribed using the High Capacity cDNA Reverse Transcription Kit and a T100™ thermal cycler. The expression of selected genes was determined by RT-PCR using the iQ Universal SYBR Green Supermix and a StepOnePlus real-time PCR equipment (Applied Biosystems). Gene expression was normalised to the housekeeping gene GAPDH and results were analyzed using the 2^−ΔΔCt^ method (n = 3, n = 3, ±SD). Statistical significance was calculated using one-way ANOVA. The specific primer sets used are listed in Table S1.

## Data Availability

Data supporting results can be found at the University of Nottingham repository (https://rdmc.nottingham.ac.uk/) and are publicly available as of the date of publication https://doi.org/10.17639/nott.7194. This paper does not report original code. Any additional information required to reanalyse the data reported in this paper is available from the lead contact upon request.

## References

[bib1] Abaci A., Guvendiren M. (2020). Designing decellularized extracellular matrix-based bioinks for 3D bioprinting. Adv. Healthc. Mater..

[bib2] Abassi Y.A., Xi B., Li N., Ouyang W., Seiler A., Watzele M., Kettenhofen R., Bohlen H., Ehlich A., Kolossov E. (2012). Dynamic monitoring of beating periodicity of stem cell-derived cardiomyocytes as a predictive tool for preclinical safety assessment. Br. J. Pharmacol..

[bib3] Agmon G., Christman K.L. (2016). Controlling stem cell behavior with decellularized extracellular matrix scaffolds. Curr. Opin. Solid State Mater. Sci..

[bib4] Alom N., Peto H., Kirkham G.R., Shakesheff K.M., White L.J. (2018). Bone extracellular matrix hydrogel enhances osteogenic differentiation of C2C12 myoblasts and mouse primary calvarial cells. J. Biomed. Mater. Res. B Appl. Biomater..

[bib5] Athukorala S.S., Tran T.S., Balu R., Truong V.K., Chapman J., Dutta N.K., Roy Choudhury N. (2021). 3D printable electrically conductive hydrogel scaffolds for biomedical applications: a review. Polymers.

[bib6] Badylak S.F., Lantz G.C., Coffey A., Geddes L.A. (1989). Small intestinal submucosa as a large diameter vascular graft in the dog. J. Surg. Res..

[bib7] Baei P., Jalili-Firoozinezhad S., Rajabi-Zeleti S., Tafazzoli-Shadpour M., Baharvand H., Aghdami N. (2016). Electrically conductive gold nanoparticle-chitosan thermosensitive hydrogels for cardiac tissue engineering. Mater. Sci. Eng. C. Mater. Biol. Appl..

[bib8] Bai R., Liu J., Zhang J., Shi J., Jin Z., Li Y., Ding X., Zhu X., Yuan C., Xiu B. (2021). Conductive single-wall carbon nanotubes/extracellular matrix hybrid hydrogels promote the lineage-specific development of seeding cells for tissue repair through reconstructing an integrin-dependent niche. J. Nanobiotechnol..

[bib9] Baumgartner S., Halbach M., Krausgrill B., Maass M., Srinivasan S.P., Sahito R.G.A., Peinkofer G., Nguemo F., Müller-Ehmsen J., Hescheler J. (2015). Electrophysiological and morphological maturation of murine fetal cardiomyocytes during electrical stimulation *in vitro*. J. Cardiovasc. Pharmacol. Ther..

[bib10] Bedada F.B., Chan S.K., Chan S.S., Metzger S.K., Zhang L., Zhang J., Garry D.J., Kamp T.J., Kyba M., Metzger J.M. (2014). Acquisition of a quantitative, stoichiometrically conserved ratiometric marker of maturation status in stem cell-derived cardiac myocytes. Stem Cell Rep..

[bib11] Casella A., Panitch A., Leach J.K. (2021). Endogenous electric signaling as a blueprint for conductive materials in tissue engineering. Bioelectricity.

[bib12] Chen Z., Chen Y., Hedenqvist M.S., Chen C., Cai C., Li H., Liu H., Fu J. (2021). Multifunctional conductive hydrogels and their applications as smart wearable devices. J. Mater. Chem. B.

[bib13] Chiti M.C., Vanacker J., Ouni E., Tatic N., Viswanath A., des Rieux A., Dolmans M.M., White L.J., Amorim C.A. (2021). Ovarian extracellular matrix-based hydrogel for human ovarian follicle survival *in vivo*: a pilot work. J. Biomed. Mater. Res. B Appl. Biomater..

[bib14] De Santis M.M., Alsafadi H.N., Tas S., Bölükbas D.A., Prithiviraj S., Da Silva I.A.N., Mittendorfer M., Ota C., Stegmayr J., Daoud F. (2021). Extracellular-matrix-reinforced bioinks for 3D bioprinting human tissue. Adv. Mater..

[bib15] Distler T., Polley C., Shi F., Schneidereit D., Ashton M.D., Friedrich O., Kolb J.F., Hardy J.G., Detsch R., Seitz H., Boccaccini A.R. (2021). Electrically conductive and 3D-printable oxidized alginate-gelatin polypyrrole: PSS hydrogels for tissue engineering. Adv. Healthc. Mater..

[bib16] Garrudo F.F.F., Nogueira D.E.S., Rodrigues C.A.V., Ferreira F.A., Paradiso P., Colaço R., Marques A.C., Cabral J.M.S., Morgado J., Linhardt R.J. (2021). Electrical stimulation of neural-differentiating iPSCs on novel coaxial electroconductive nanofibers. Biomater. Sci..

[bib17] Goding J.A., Gilmour A.D., Aregueta-Robles U.A., Hasan E.A., Green R.A. (2018). Living bioelectronics: strategies for developing an effective long-term implant with functional neural connections. Adv. Funct. Mater..

[bib18] Grune T., Ott C., Häseli S., Höhn A., Jung T. (2019). The “MYOCYTER”–Convert cellular and cardiac contractions into numbers with ImageJ. Sci. Rep..

[bib19] Hassarati R.T., Dueck W.F., Tasche C., Carter P.M., Poole-Warren L.A., Green R.A. (2014). Improving cochlear implant properties through conductive hydrogel coatings. IEEE Trans. Neural Syst. Rehabil. Eng..

[bib20] Herrmann A., Haag R., Schedler U. (2021). Hydrogels and their role in biosensing applications. Adv. Healthc. Mater..

[bib21] Hinton T.J., Jallerat Q., Palchesko R.N., Park J.H., Grodzicki M.S., Shue H.-J., Ramadan M.H., Hudson A.R., Feinberg A.W. (2015). Three-dimensional printing of complex biological structures by freeform reversible embedding of suspended hydrogels. Sci. Adv..

[bib22] Hwang J., San B.H., Turner N.J., White L.J., Faulk D.M., Badylak S.F., Li Y., Yu S.M. (2017). Molecular assessment of collagen denaturation in decellularized tissues using a collagen hybridizing peptide. Acta Biomater..

[bib23] Imaninezhad M., Pemberton K., Xu F., Kalinowski K., Bera R., Zustiak S.P. (2018). Directed and enhanced neurite outgrowth following exogenous electrical stimulation on carbon nanotube-hydrogel composites. J. Neural Eng..

[bib24] Izadifar M., Chapman D., Babyn P., Chen X., Kelly M.E. (2018). UV-assisted 3D bioprinting of nanoreinforced hybrid cardiac patch for myocardial tissue engineering. Tissue Eng. Part C Methods.

[bib25] Jang J., Kim T.G., Kim B.S., Kim S.-W., Kwon S.-M., Cho D.-W. (2016). Tailoring mechanical properties of decellularized extracellular matrix bioink by vitamin B2-induced photo-crosslinking. Acta Biomater..

[bib26] Joung D., Truong V., Neitzke C.C., Guo S.Z., Walsh P.J., Monat J.R., Meng F., Park S.H., Dutton J.R., Parr A.M., McAlpine M.C. (2018). Spinal cord scaffolds: 3D printed stem-cell derived neural progenitors generate spinal cord scaffolds. Adv. Funct. Mater..

[bib27] Kim B.S., Das S., Jang J., Cho D.-W. (2020). Decellularized extracellular matrix-based bioinks for engineering tissue-and organ-specific microenvironments. Chem. Rev..

[bib28] Lam D., Enright H.A., Peters S.K., Moya M.L., Soscia D.A., Cadena J., Alvarado J.A., Kulp K.S., Wheeler E.K., Fischer N.O. (2020). Optimizing cell encapsulation condition in ECM-Collagen I hydrogels to support 3D neuronal cultures. J. Neurosci. Methods.

[bib29] Leonard A., Bertero A., Powers J.D., Beussman K.M., Bhandari S., Regnier M., Murry C.E., Sniadecki N.J. (2018). Afterload promotes maturation of human induced pluripotent stem cell derived cardiomyocytes in engineered heart tissues. J. Mol. Cell. Cardiol..

[bib30] Li X.-P., Qu K.-Y., Zhou B., Zhang F., Wang Y.-Y., Abodunrin O.D., Zhu Z., Huang N.-P. (2021). Electrical stimulation of neonatal rat cardiomyocytes using conductive polydopamine-reduced graphene oxide-hybrid hydrogels for constructing cardiac microtissues. Colloids Surf. B Biointerfaces.

[bib31] Liu X., Miller A.L., Park S., Waletzki B.E., Zhou Z., Terzic A., Lu L. (2017). Functionalized carbon nanotube and graphene oxide embedded electrically conductive hydrogel synergistically stimulates nerve cell differentiation. ACS Appl. Mater. Interfaces.

[bib32] Loneker A.E., Faulk D.M., Hussey G.S., D'Amore A., Badylak S.F. (2016). Solubilized liver extracellular matrix maintains primary rat hepatocyte phenotype in-vitro. J. Biomed. Mater. Res. A.

[bib33] Maiullari F., Costantini M., Milan M., Pace V., Chirivì M., Maiullari S., Rainer A., Baci D., Marei H.E.-S., Seliktar D. (2018). A multi-cellular 3D bioprinting approach for vascularized heart tissue engineering based on HUVECs and iPSC-derived cardiomyocytes. Sci. Rep..

[bib34] Mawad D., Lauto A., Wallace G.G. (2016). Polymeric Hydrogels as Smart Biomaterials.

[bib35] Mosqueira D., Mannhardt I., Bhagwan J.R., Lis-Slimak K., Katili P., Scott E., Hassan M., Prondzynski M., Harmer S.C., Tinker A. (2018). CRISPR/Cas9 editing in human pluripotent stem cell-cardiomyocytes highlights arrhythmias, hypocontractility, and energy depletion as potential therapeutic targets for hypertrophic cardiomyopathy. Eur. Heart J..

[bib36] Ouyang L., Yao R., Zhao Y., Sun W. (2016). Effect of bioink properties on printability and cell viability for 3D bioplotting of embryonic stem cells. Biofabrication.

[bib37] Pati F., Jang J., Ha D.-H., Won Kim S., Rhie J.-W., Shim J.-H., Kim D.-H., Cho D.-W. (2014). Printing three-dimensional tissue analogues with decellularized extracellular matrix bioink. Nat. Commun..

[bib38] Qin C., Yue Z., Chao Y., Forster R.J., Maolmhuaidh F.Ó., Huang X.-F., Beirne S., Wallace G.G., Chen J. (2020). Bipolar electroactive conducting polymers for wireless cell stimulation. Appl. Mater. Today.

[bib39] Rastin H., Zhang B., Mazinani A., Hassan K., Bi J., Tung T.T., Losic D. (2020). 3D bioprinting of cell-laden electroconductive MXene nanocomposite bioinks. Nanoscale.

[bib40] Ravanbakhsh H., Bao G., Latifi N., Mongeau L.G. (2019). Carbon nanotube composite hydrogels for vocal fold tissue engineering: biocompatibility, rheology, and porosity. Mater. Sci. Eng. C. Mater. Biol. Appl..

[bib41] Ravi S., Caves J.M., Martinez A.W., Xiao J., Wen J., Haller C.A., Davis M.E., Chaikof E.L. (2012). Effect of bone marrow-derived extracellular matrix on cardiac function after ischemic injury. Biomaterials.

[bib42] Saldin L.T., Cramer M.C., Velankar S.S., White L.J., Badylak S.F. (2017). Extracellular matrix hydrogels from decellularized tissues: structure and function. Acta Biomater..

[bib43] Sánchez E.M., Gómez-Blanco J.C., López Nieto E., Casado J.G., Macías-García A., Díaz Díez M.A., Carrasco-Amador J.P., Torrejón Martín D., Sánchez-Margallo F.M., Pagador J.B. (2020). Hydrogels for bioprinting: a systematic review of hydrogels synthesis, bioprinting parameters, and bioprinted structures behavior. Front. Bioeng. Biotechnol..

[bib44] Sanjuan-Alberte P., Vaithilingam J., Moore J.C., Wildman R.D., Tuck C.J., Alexander M.R., Hague R.J.M., Rawson F.J. (2021). Development of conductive gelatine-methacrylate inks for two-photon polymerisation. Polymers.

[bib45] Sawkins M.J., Bowen W., Dhadda P., Markides H., Sidney L.E., Taylor A.J., Rose F.R., Badylak S.F., Shakesheff K.M., White L.J. (2013). Hydrogels derived from demineralized and decellularized bone extracellular matrix. Acta Biomater..

[bib46] Shin Y.J., Shafranek R.T., Tsui J.H., Walcott J., Nelson A., Kim D.-H. (2021). 3D bioprinting of mechanically tuned bioinks derived from cardiac decellularized extracellular matrix. Acta Biomater..

[bib47] Soltan N., Ning L., Mohabatpour F., Papagerakis P., Chen X. (2019). Printability and cell viability in bioprinting alginate dialdehyde-gelatin scaffolds. ACS Biomater. Sci. Eng..

[bib48] Spencer A.R., Shirzaei Sani E., Soucy J.R., Corbet C.C., Primbetova A., Koppes R.A., Annabi N. (2019). Bioprinting of a cell-laden conductive hydrogel composite. ACS Appl. Mater. Interfaces.

[bib49] Toeg H.D., Tiwari-Pandey R., Seymour R., Ahmadi A., Crowe S., Vulesevic B., Suuronen E.J., Ruel M. (2013). Injectable small intestine submucosal extracellular matrix in an acute myocardial infarction model. Ann. Thorac. Surg..

[bib50] Tsui J.H., Leonard A., Camp N.D., Long J.T., Nawas Z.Y., Chavanachat R., Smith A.S., Choi J.S., Dong Z., Ahn E.H. (2021). Tunable electroconductive decellularized extracellular matrix hydrogels for engineering human cardiac microphysiological systems. Biomaterials.

[bib51] Uzun A.U., Mannhardt I., Breckwoldt K., Horváth A., Johannsen S.S., Hansen A., Eschenhagen T., Christ T. (2016). Ca2+-currents in human induced pluripotent stem cell-derived cardiomyocytes effects of two different culture conditions. Front. Pharmacol..

[bib52] Vaithilingam J., Sanjuan-Alberte P., Campora S., Rance G.A., Jiang L., Thorpe J., Burroughs L., Tuck C.J., Denning C., Wildman R.D. (2019). Multifunctional bioinstructive 3D architectures to modulate cellular behavior. Adv. Funct. Mater..

[bib53] Voytik-Harbin S.L., Brightman A.O., Waisner B.Z., Robinson J.P., Lamar C.H. (1998). Small intestinal submucosa: a tissue-derived extracellular matrix that promotes tissue-specific growth and differentiation of cells *in vitro*. Tissue Eng..

[bib54] Wang S., Guan S., Xu J., Li W., Ge D., Sun C., Liu T., Ma X. (2017). Neural stem cell proliferation and differentiation in the conductive PEDOT-HA/Cs/Gel scaffold for neural tissue engineering. Biomater. Sci..

[bib55] Wang Y.L., Han L., Zhang X.L., Cao L., Hu K., Li L.H., Wei Y. (2021). 3D bioprinting of an electroactive and self-healing polysaccharide hydrogels. J. Tissue Eng. Regen. Med..

[bib56] White L.J., Taylor A.J., Faulk D.M., Keane T.J., Saldin L.T., Reing J.E., Swinehart I.T., Turner N.J., Ratner B.D., Badylak S.F. (2017). The impact of detergents on the tissue decellularization process: a ToF-SIMS study. Acta Biomater..

