# Supplementary material for: Printing biohybrid materials for bioelectronic cardio-3D-cellular constructs
Source: iScience. 2022 Jun 7;25(7):104552. doi: 10.1016/j.isci.2022.104552 (PMC9240791; doi:10.1016/j.isci.2022.104552)
Supplement: Document S1. Figures S1–S12 and Table S1 [file mmc1.pdf]

iScience, Volume *25*

Supplemental information

Printing biohybrid materials

for bioelectronic cardio-3D-cellular constructs

Paola Sanjuan-Alberte, Charlie Whitehead, Joshua N. Jones, João C. Silva, Nathan Carter, Simon Kellaway, Richard J.M. Hague, Joaquim M.S. Cabral, Frederico C. Ferreira, Lisa J. White, and Frankie J. Rawson

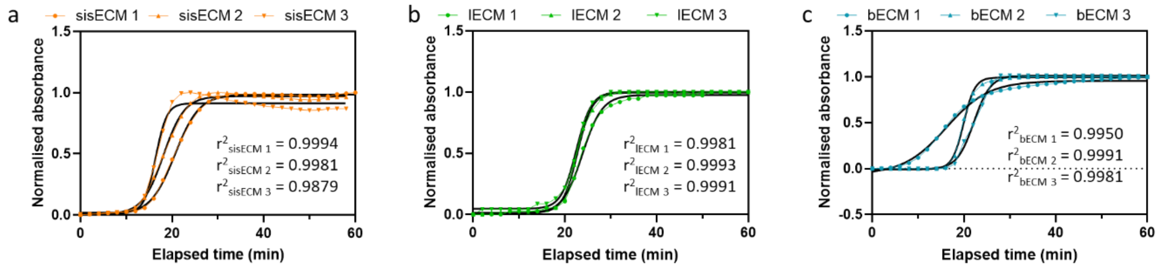

Figure S1. Fitting of sigmoidal curves from the normalised absorbance values obtained during the gel kinetics determination of (a) sisECM, (b) lECM and (c) bECM at 450 nm. Related to Figure 2.

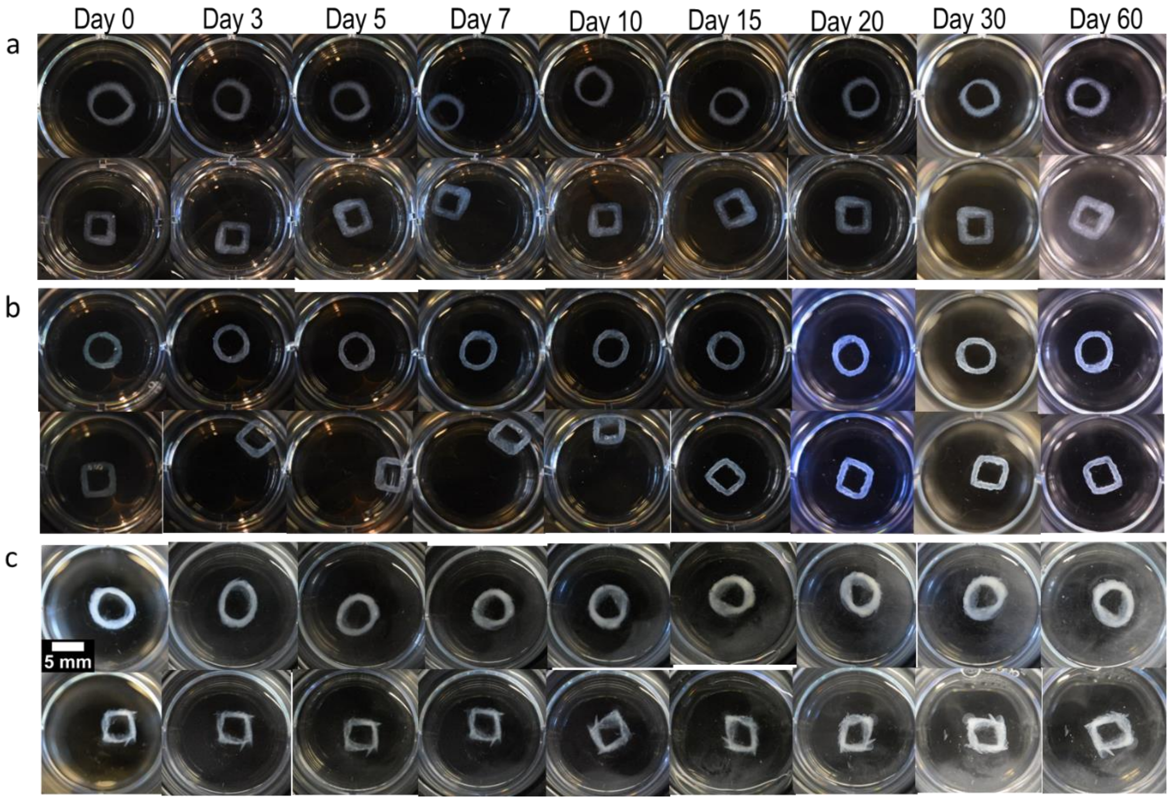

Figure S2. Stability of FRESH extruded **(a)** sisECM, **(b)** lECM and **(c)** bECM structures over time. These structures consisted on 6 mm diameter rings and squares with 6 mm sides. Related to Figure 2.

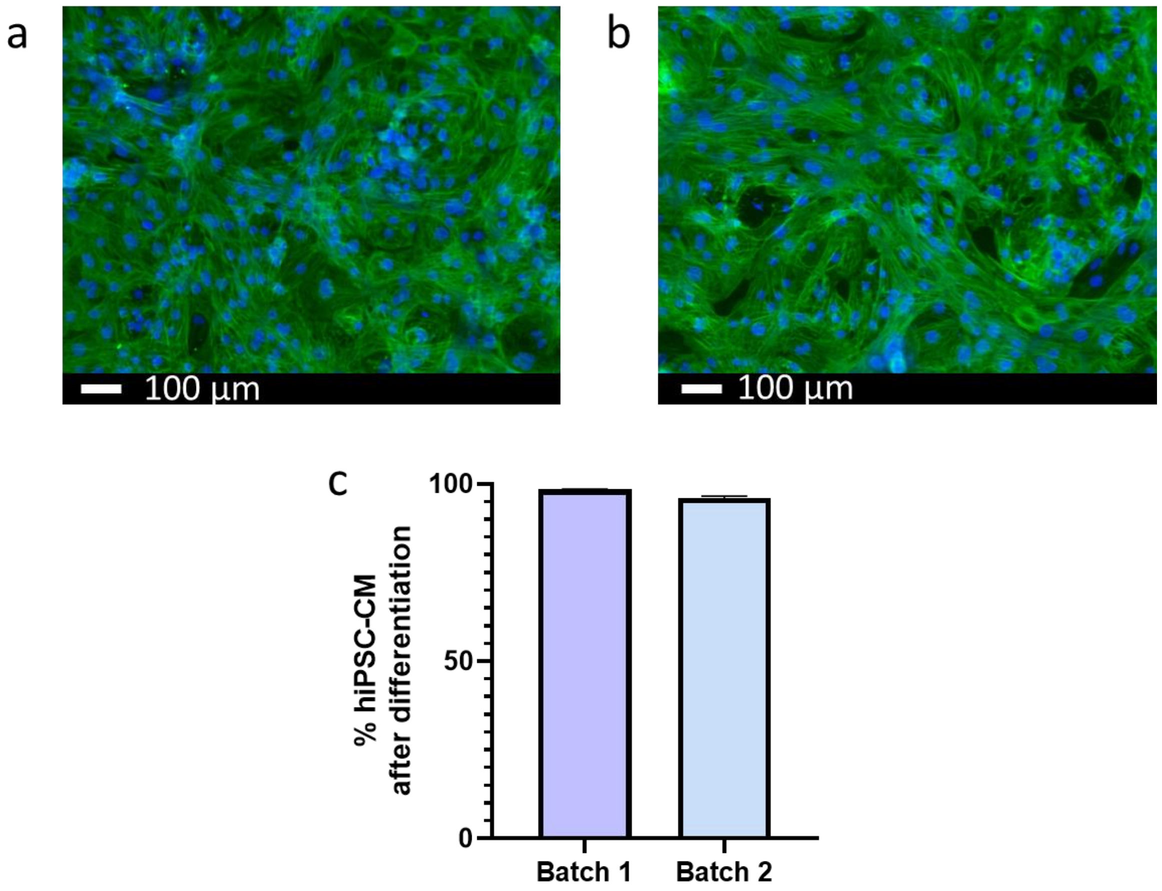

Figure S3. Immunostaining of two different batches **(a,b)** of hPSC-CMs after differentiation. Representative images were selected. Cells were immunolabelled for cardiac troponin TNNI3 (green). Nuclei were counterstained with Hoechst (blue). hPSC-CMs are positive for the green markers, whereas other cell types are stained only with Hoechst. **(c)** Determination of hPSC-CMs purity of the differentiated batches. Related to Figure 2.

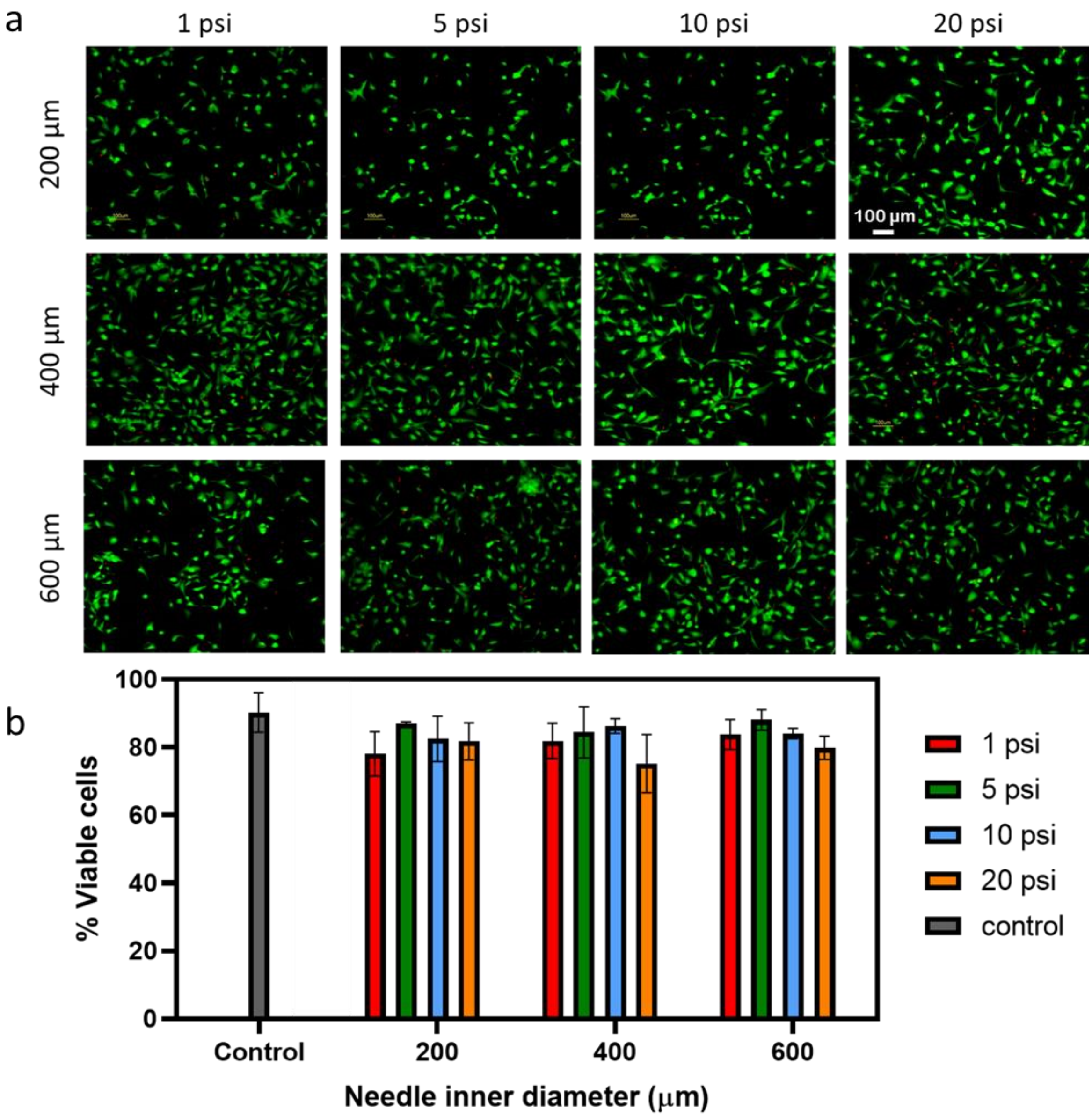

Figure S4. (a) Live/Dead staining of human pluripotent stem cell derived cardiomyocytes (hPSC-CMs) 24 hours after bioprinting using various inner needle diameters and pressures. Scale bar 100 µm. **(b)** Calculated percentage of viable cells for each condition (n=3, ±SD). Related to Figure 2.

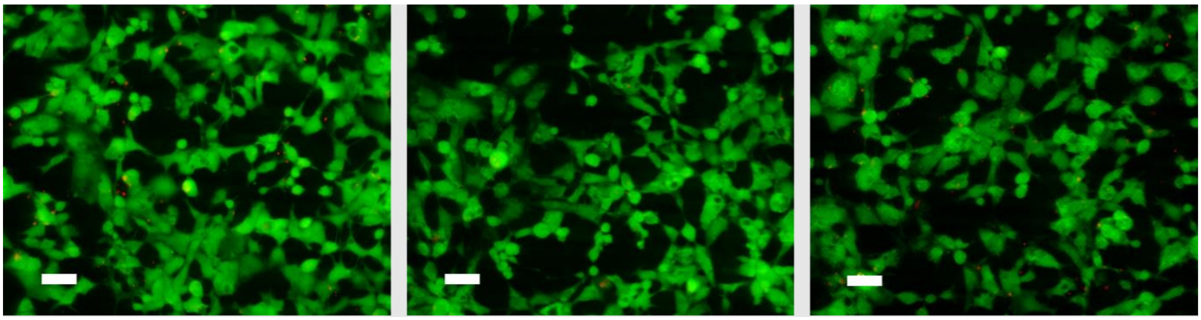

Figure S5. Live/Dead staining of hPSC-CMs in three different samples cultured on control surfaces (Well-plate). Scale bar 50 µm. Related to Figure 2.

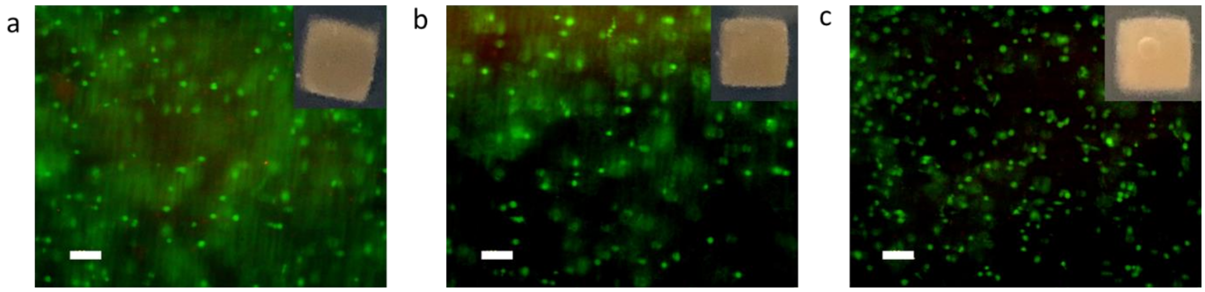

Figure S6. Live/Dead staining of hPSC-CMs encapsulated on **(a)** sisECM+MWCNTs 1 mg ml^{-1}, **(b)** lECM+MWCNTs 1 mg ml^{-1}, **(c)** bECM+MWCNTs 1 mg ml^{-1} and inset images of the different hydrogels. Scale bar 100 µm. Related to Figure 3.

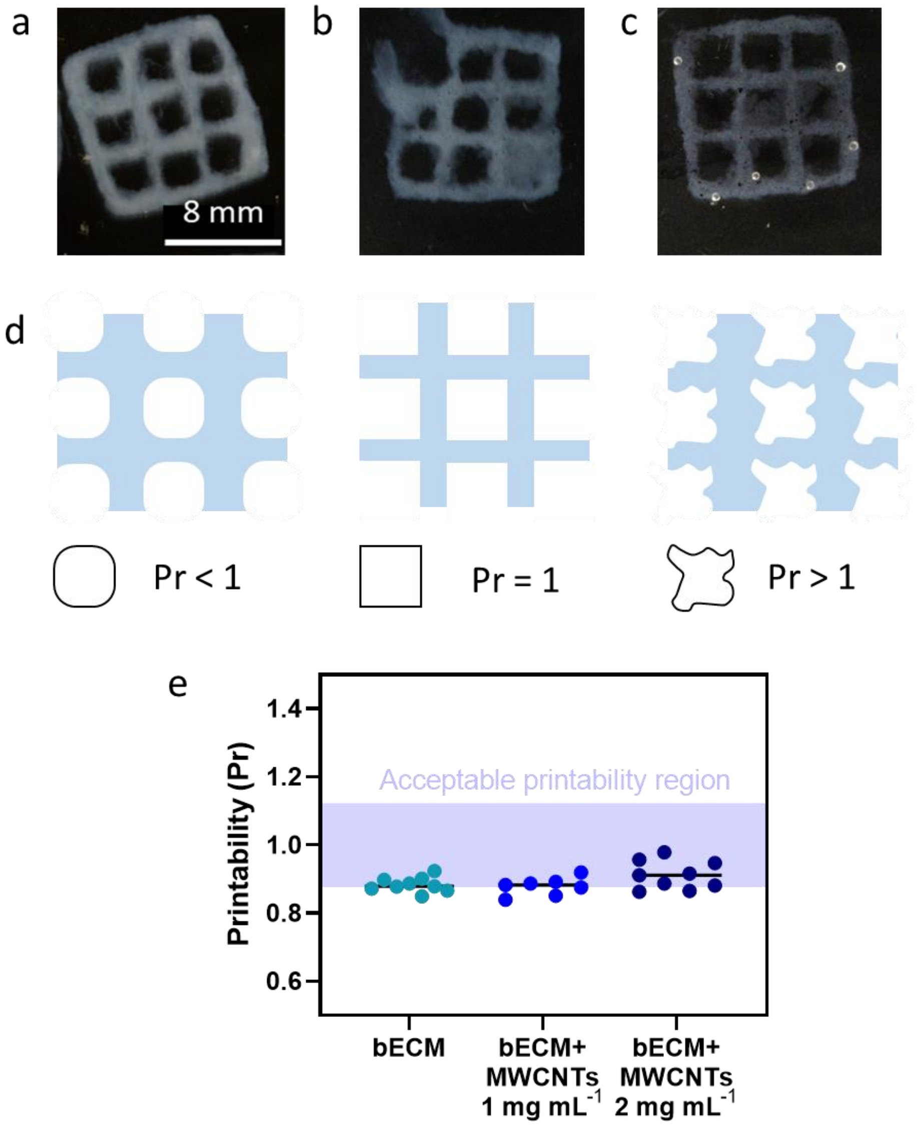

Figure S7. Printed 10 mm square scaffold using (a) bECM, (b) bECM+MWCNTs 1 mg mL-1 and (c) bECM+MWCNTs 2 mg mL-1. (d) Evaluation of printability (Pr) under three typical conditions. (e) Semi-quantified Pr value of printed constructs. Related to Figure 4.

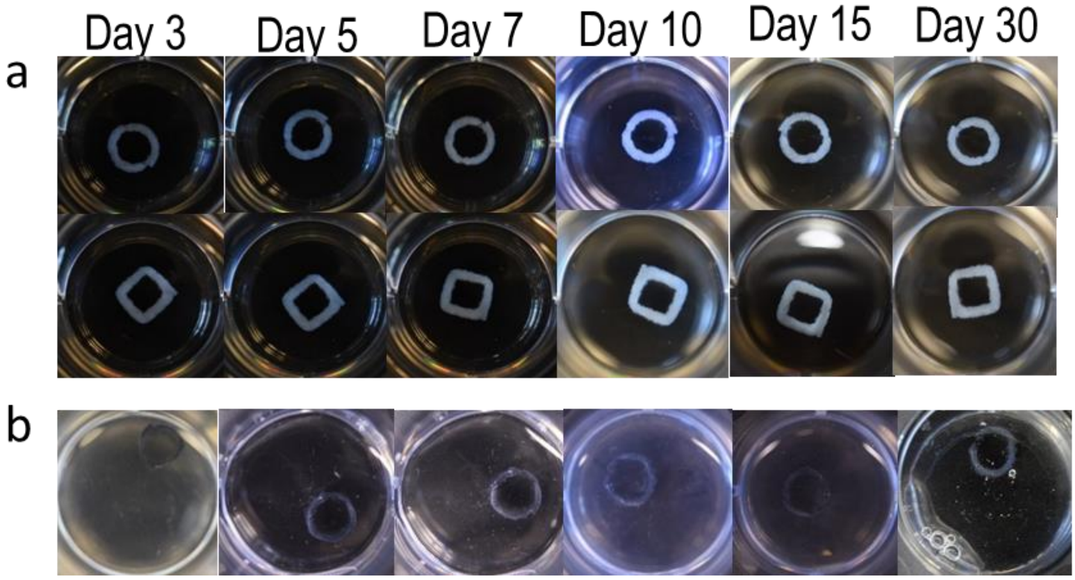

Figure S8. Stability of FRESH extruded bECM at **(a)** 1 mg mL^{-1} and **(b)** 2 mg mL^{-1} multi-walled carbon nanotubes (MWCNTs) concentration. Related to Figure 4.

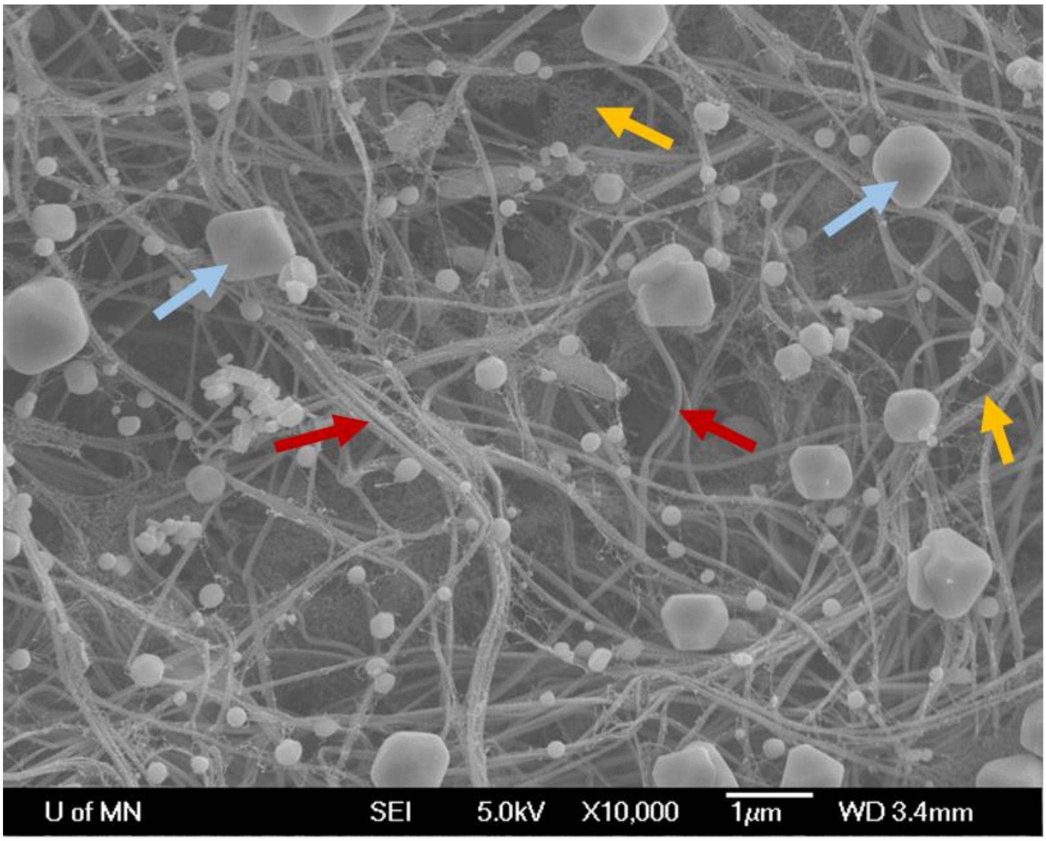

Figure S9. SEM images of bECM and MWCNTs at a concentration of 0.2 mg mL^{-1} the different elements can be observed: collagen fibres (red arrows), MWCNTs (yellow arrows) and buffer salts (blue arrows). Related to Figure 4.

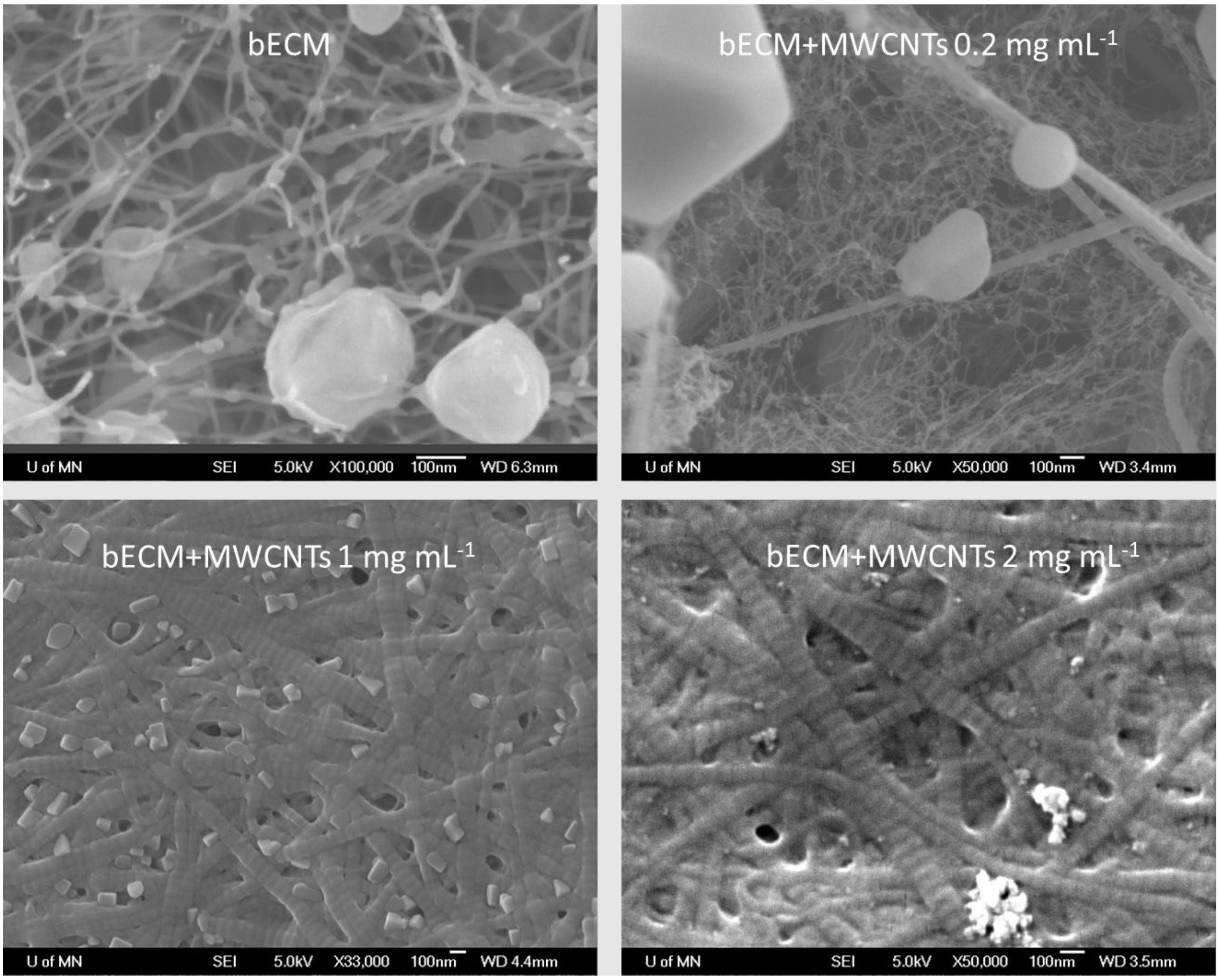

Figure S10. SEM images at higher magnifications. Related to Figure 4.

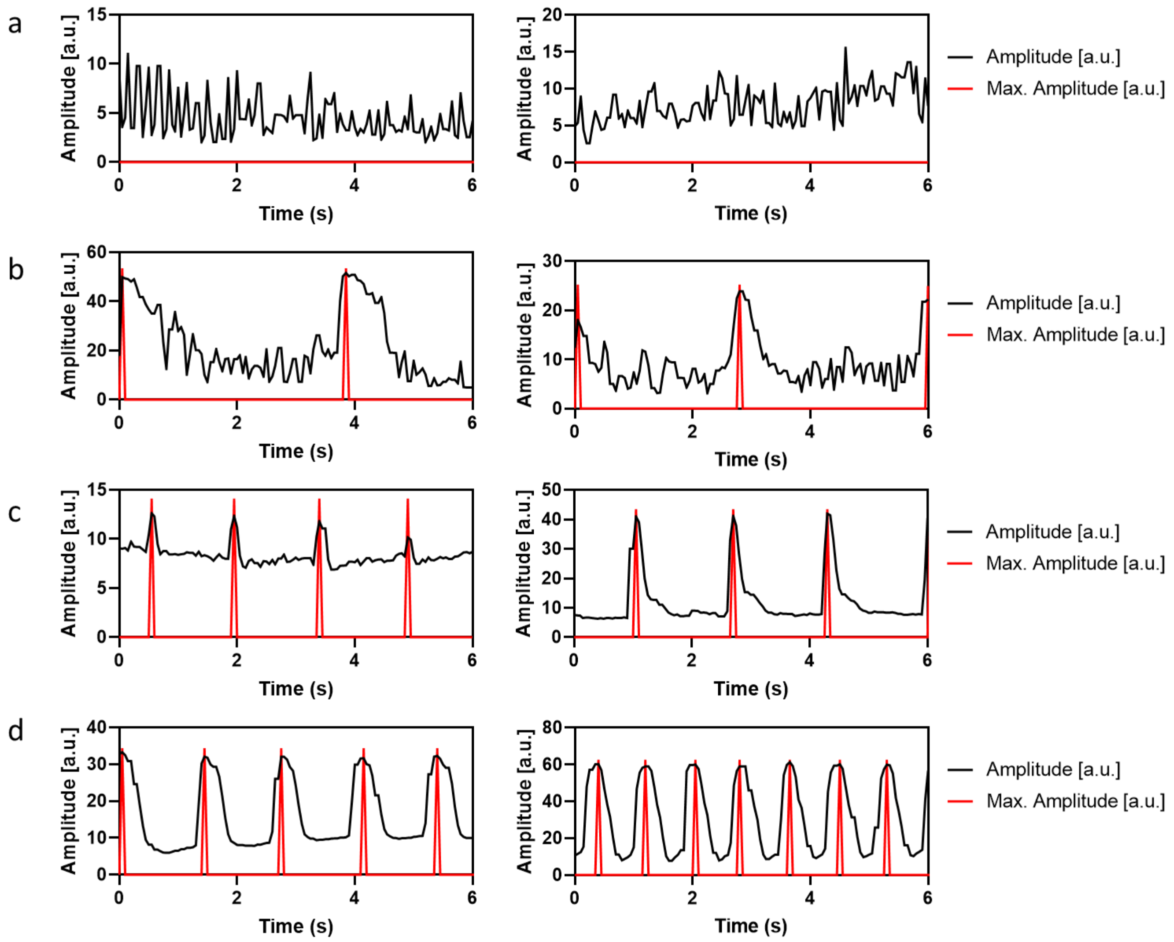

Figure S11. Time-dependent changes in autonomous contractile behaviour of hPSC-CMs of additional replicates determined using the analytical tool Myocyter (v1.3) of (a) bECM, (b) bECM+MWCNTs 1mg mL-1, (c) bECM under electrical stimulation (ES) and (d) bECM+MWCNTs 1mg mL-1 under ES. Related to Figure 5.

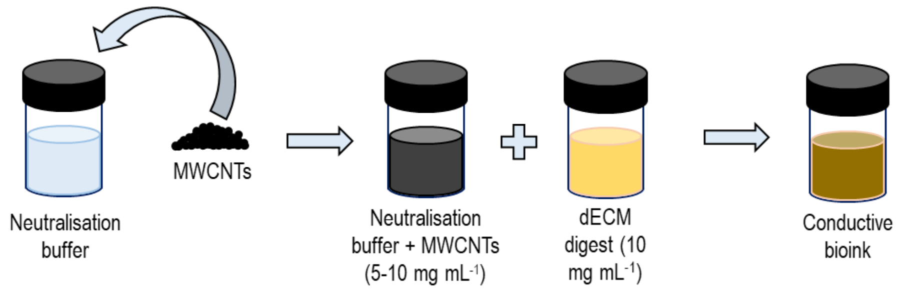

Figure S12. Schematic showing the procedure for preparation of the conductive bioinks Related to STAR Methods

Table S1. List of primers used. Related to STAR Methods

Gene		Sequence	Size (bp)
cTNNI (TNNI3)	Forward	CCTCCAACTACCGCGCTTAT	20
	Reverse	CTGCAATTTTCTCGAGGCGG	20
ssTNNI (TNNI1)	Forward	GCTCCACGAGGACTGAACAA	20
	Reverse	CTTCAGCAAGAGTTTGCGGG	20
TTN-2NB	Forward	CCAATGAGTATGGCAGTGTCA	21
	Reverse	TACGTTCCGGAAGTAATTTGC	21
SERCA2	Forward	ACCCACATTCGAGTTGGAAG	20
	Reverse	CCAACGAAGGTCAGATTGGT	20
RYR2	Forward	AAGCCCTCTCGTCTGAAACA	20
	Reverse	CCACCCAGACATTAGCAGGT	20
CACNA1C	Forward	CAATCTCCGAAGAGGGGTTT	20
	Reverse	TCGCTTCAGACATTCCAGGT	20
CACNA1H	Forward	TCAACGTCATCACCATGTCC	20
	Reverse	AGCCTCGAAGACAAACACGA	20
GAPDH	Forward	AACAGCGACACCCACTCCTC	20
	Reverse	CATACCAGGAAATGAGCTTGACAA	24
